# A novel systematic byte substitution method to design strong bijective substitution box (S-box) using piece-wise-linear chaotic map

**DOI:** 10.7717/peerj-cs.940

**Published:** 2022-05-11

**Authors:** Asim Ali, Muhammad Asif Khan, Ramesh Kumar Ayyasamy, Muhammad Wasif

**Affiliations:** 1 Computer Science, Comsats University Islamabad, Wah Cantt Campus, Punjab, Pakistan; 2 Computer Science, University of Wah, Wah Cantt, Punjab, Pakistan; 3 Computer Engineering Department, University of Engineering and Technology Taxila, Taxila, Punjab, Pakistan; 4 Department of Information Systems, Faculty of Information and Communication Technology, Universiti Tunku Abdul Rahman (UTAR), Kampar, Perak, Malaysia; 5 Department of Computer Science, Comsats University Islamabad, Wah Cantt Campus, Punjab, Pakistan

**Keywords:** Chaos, Cryptography, Differential probability, Substitution-box

## Abstract

Cryptography deals with designing practical mathematical algorithms having the two primitive elements of confusion and diffusion. The security of encrypted data is highly dependent on these two primitive elements and a key. S-box is the nonlinear component present in a symmetric encryption algorithm that provides confusion. A cryptographically strong bijective S-box structure in cryptosystem ensures near-optimal resistance against cryptanalytic attacks. It provides uncertainty and nonlinearity that ensures high confidentiality and security against cryptanalysis attacks. The nonlinearity of an S-box is highly dependent on the dispersal of input data using an S-box. Cryptographic performance criteria of chaos-based S-boxes are worse than algebraic S-box design methods, especially differential probability. This article reports a novel approach to design an 8 × 8 S-box using chaos and randomization using dispersion property to S-box cryptographic properties, especially differential probability. The randomization using dispersion property is introduced within the design loop to achieve low differential uniformity possibly. Two steps are involved in generating the proposed S-box. In the first step, a piecewise linear chaotic map (PWLCM) is utilized to generate initial S-box positions. Generally, the dispersion property is a post-processing technique that measures maximum nonlinearity in a given random sequence. However, in the second step, the concept is carefully reverse engineered, and the dispersion property is used within the design loop for systematic dispersal of input substituting sequence. The proposed controlled randomization changes the probability distribution statistics of S-box’s differentials. The proposed methodology systematically substitutes the S-box positions that cause output differences to recur for a given input difference. The proposed S-box is analyzed using well-established and well-known statistical cryptographic criteria of nonlinearity, strict avalanche criteria (SAC), bit independence criteria (BIC), differential probability, and linear probability. Further, the S-box’s boomerang connectivity table (BCT) is generated to analyze its strength against boomerang attack. Boomerang is a relatively new attacking framework for cryptosystem. The proposed S-box is compared with the state-of-the-art latest related publications. Results show that the proposed S-box achieves an upper bound of cryptographic properties, especially differential probability. This work hypothesizes that highly dispersive hamming distances at output difference, generated a systematic S-box. The mixing property of chaos generated trajectories utilized for decimal mapping. To test the randomness of generated chaotic trajectories, a cryptographically secure pseudo-random sequence was generated using a chaotic map that was tested using the National Institute of Standards and Technology (NIST) NIST-800-22 test suit.

## Introduction

Cryptography aids individual users and corporate organizations in protecting their digital data and information. With the prevalence of cryptography (Paar & Pelzl, 2009), digital data transmission over an insecure network has significantly improved. This rapid increase in transmission has entailed a significant enhancement of information security. The new standards for data communication and information technology have developed with the requirement of a specific mechanism to resist cryptographic attacks (Standaert, Piret & Quisquater, 2003; National Institute of Standards and Technology, 2001; Biryukov, 2011; Daemen & Rijmen, 2002). With his paper on the communication theory of secrecy, Shannon has laid the foundation of a modern era of cryptography (Shannon, 1949). Symmetric and asymmetric critical cryptographic algorithms at the byte/word level or bit-level are used to secure and protect digital information transmitted over insecure channels. In the light of the previous discussion, this paper attempts to design a systematic S-box with improved cryptographic property, especially DP.

Data confidentially in cryptography is related to the encryption of digital data. Modern block ciphers, including DES (National Institute of Standards & Technology, 1999) and variants of DES, Blowfish (Schneier, 1993), Camelia (Aoki et al., 2001), Kasumi (ETSI, 2001), RC5 (Rivest, 1995), RC6 (Handschuh, 2011), PRESENT (Bogdanov et al., 2007), and AES (Daemen & Rijmen, 2002) are based on Shannon principle of confusion and diffusion. Confusion is a technique that obscures the relationship between the key and the ciphertext, thus making it difficult for an attacker to guess the key while wiretapping. An S-box, a nonlinear auxiliary table, is used in the encryption algorithm as a confusion component.

An S-box is a bijective mapping $S =\{ (0,1)^n \mapsto (0,1)^n\}$ where equality exhibits that input and output bits are the same, hence an asymmetric S-box. An S-box ensures nonlinear propagation of plaintext through rounds of an encryption algorithm to achieve confusion and prevent an attacker from recovering the correct key. After introducing differential cryptanalysis (Biham & Shamir, 1991; Langfordl & Hellman, 1994), an expanded set of S-box design criteria was proposed (Dawson & Tavares, 1991; Yi, Cheng & You, 1997; Nyberg, 1991). It was revealed in the early ‘90s that the known structure acts as a basis to mount the differential cryptanalysis. Therefore, an S-box based on given criteria preferably leads to near-optimal resistance against differential and linear attacks.

Differential cryptanalysis is a beneficial attack on block ciphers, also known as a chosen-plaintext attack. To mount this attack, a cryptanalyst first chooses input differential $\Delta x$ of plaintext pairs (x, x′), examines the propagation, and finds output differential pairs through encryption. In this attack, a cryptanalyst uses an S-box to compute a complete set of output differences (∆y) for all given input differences (∆x). Subsequently, input/output differences are tabulated as a difference distribution table (DDT). It searches for high probable output pairs for a given $\Delta x$ through differential analysis of a cipher. Thus, a differential attack marks weaknesses within the cipher and achieves desirable results on the part of an attacker (Heys, 2002; Biham & Shamir, 1991). The following definitions will help understand the concept of DDT to measure DP (Biryukov & Perrin, 2015; Biham & Shamir, 1991).

**Definition 1:** Let ${\rm S} =\{ (0,1)^{\rm n} \mapsto (0,1)^{\rm m}\}$ where “ $({\rm{m}} = {\rm{n}})$”, is a substitution function. The number of pairs gives the DP of the differential (∆*x*, ∆*y*) with input difference $\Delta x$ and output difference $\Delta y$, divided by the total number of pairs with input difference $\Delta {\rm x}$

(1) $$D{P_{\left( {\Delta x,\Delta y} \right)}} = \# \left\{ {x \in X|S\left( x \right) \oplus S\left( {x \oplus \Delta x} \right) = \Delta y} \right\}/{2^n}$$

The DP is considered a stochastic variable and can only take limited values of either 0 or multiple of ${2^{1 - n}}$.

Rijndael S-box was a two-step algebraic design based on AES’s GF(256) inverse and affine transformation. It was based on the NIST criteria, inspired by the linear and differential attack (Biham & Shamir, 1991; Matsui, 1996). The introduction of AES established the basis to design strong cryptosystems. In the same era, Kocarev (2001) portrays an excellent foundation on chaos-based cryptography and summarizes similarities and differences between a chaotic map and cryptographic algorithms. For example, chaotic maps are defined on a subset of real numbers, and cryptographic algorithms are defined on finite sets. The parameters of a map may represent the key of an encryption algorithm. Encryption rounds in a cryptographic algorithm fulfill the desired confusion and diffusion properties, and the iterations of a chaotic map fulfill the ergodicity property. Chaos has deterministic dynamics and has properties like positive Lyapunov exponent, mixing, and ergodicity. These properties are favored in cryptography and have an advantage over algebraic designs due to their less computational complexity, ease of implementation, sensitive dependence on initial conditions, and hardware efficiency.

### Related literature on chaos-based S-box

An extensive research has been presented in literature utilizing chaotic maps in various domains, such as digital image encryption (Pareek, Patidar & Sud, 2006; Murillo-Escobar et al., 2015; Yavuz et al., 2016; Liu et al., 2015; Wang et al., 2019; Farwa et al., 2017), watermarking (Jamal, Khan & Shah, 2016; Khan et al., 2019; Singh & Raman, 2017), steganography (EL-Latif, Abd-El-Atty & Venegas-Andraca, 2019; Siddiqui & Khare, 2021; Pak et al., 2019), Light-Weight cryptography and IoT (Mondal & Mandal, 2017; Mohananthini, Mohamed Parvees & Abdul Samath, 2021; Prathiba & Bhaaskaran, 2018), cryptographically-secure random number generation (Behnia et al., 2011; Avaroʇlu et al., 2014; Rezk et al., 2019; Koyuncu & Turan Özcerit, 2017; Irfan et al., 2020) and healthcare applications (Rajendran & Doraipandian, 2021; Masood et al., 2021; Magsi et al., 2021). A few novel methodologies have been proposed to design an S-box using the mixing property of the chaotic map (Jakimoski & Kocarev, 2001; Tang, Liao & Chen, 2005). Jakimoski & Kocarev (2001) proposed S-boxes using the chaotic logistic map. Their methodology was a four-step process utilizing a chaotic logistic map. Tang, Liao & Chen (2005) proposed a two-step 8 × 8 S-box methodology by iterating a chaotic map. First, the chaotic map is iterated to obtain distinct integers in the range (0 – 2^*n*^) are stored in an integer table. Secondly, a 2D Baker map permutes the integer table to obtain the final S-box. Later on, many researchers showed their potential and improved chaos-based S-boxes utilizing 1-dimensional chaotic maps (Belazi & El-Latif, 2017; Lambić, 2018; Shakiba, 2020; Tanyildizi & Özkaynak, 2019), higher-dimensional maps (Özkaynak, 2020; 2017; Özkaynak, Çelik & Özer, 2017; Lu, Zhu & Wang, 2019; Tian & Lu, 2016; Chen, Chen & Liao, 2007; Tang, Liao & Chen, 2005; Chen et al., 2008), and hybrid techniques incorporating optimization techniques with chaos, such as genetic algorithm (Li-Jiang & Tian-Lun, 2002; Wang et al., 2020), Hill climbing (Alzaidi et al., 2018), firefly algorithm (Ahmed, Zolkipli & Ahmad, 2019), heuristic techniques (Ahmad, Alauddin & AlSharari, 2018; Ahmad et al., 2020; Farah, Rhouma & Belghith, 2017). Few S-box design methodologies exploited cellular automata (Seredynski, Bouvry & Zomaya, 2004; Szaban & Seredynski, 2012; Miroslaw & Seredynski, 2011; Picek et al., 2017; Gangadari et al., 2015), elliptic curve (Hayat, Azam & Asif, 2018; Azam, Hayat & Ullah, 2018) for comparable properties of S-box design.

Recently, research on the S-box has been accelerated and numerous methodologies have been proposed (Ahmed, Zolkipli & Ahmad, 2019; Açikkapi, Özkaynak & Özer, 2019; Hussain et al., 2019; Lu, Zhu & Wang, 2019; Zahid, Arshad & Ahmad, 2019; Khan, Ahmed & Saleem, 2019; Farhan et al., 2019; Yi et al., 2019; Özkaynak, 2019; Tanyildizi & Özkaynak, 2019; Özkaynak, 2020; Artuğer & Özkaynak, 2020; Shakiba, 2020; Zhu et al., 2020; Dimitrov, 2020; Nizam Chew & Ismail, 2020; Ahmad et al., 2020; Wang et al., 2020; Faheem et al., 2020; Alhadawi et al., 2021; Khan & Jamal, 2021; Zamli, 2021; Zahid et al., 2021; Hua et al., 2021; Jiang & Ding, 2021; Belazi & El-Latif, 2017). Belazi & El-Latif (2017) proposed a four-step S-box design methodology based on a chaotic sine map. In step 1, the integer matrix S(16 × 16) is obtained. Steps 2 and 4 are the reshaping function to obtain row vector of I(1 × 256). Step 3 utilizes a chaotic sine map to generate a permutation of integer matrix S(16 × 16). In Tian & Lu, 2017 a method based on a 1-D logistic map and optimized using the bacterial foraging optimization method was proposed. An algebraic method using cubic traction transform was proposed (Zahid, Arshad & Ahmad, 2019). Wang et al. (2020) proposed an S-box design method based on a logistic map and genetic algorithm. The proposed methodology is two-step. First, a chaotic logistic map generates the initial pool of S-boxes. Secondly, a genetic algorithm is applied to obtain the final S-box. Shakiba (2020) proposed a simple chaotic S-box based on the I-D Chebyshev map. Artuğer & Özkaynak (2020) proposed a method to analyze chaotic S-box design using the zigzag mapping technique. Various discrete and continuous maps are chosen, and integer mapping is performed using the zigzag transformation approach. In Ahmad et al. (2020) a hybrid approach to design a bijective S-box was proposed. First, key-dependent improved S-boxes are generated using I-D sine-powered chaotic map and heuristic search technique. Secondly, chaotic features of obtained S-boxes are improvised using the action of an algebraic group. In Khan & Jamal (2021) author proposed an S-box design based on the composition of chaotic maps for lightweight design. In Zahid et al. (2021) a method to design S-box based on heuristic evolutionary strategy and modular operation is presented. Hua et al. (2021) proposed an S-box method using an improved logistic map and bijective matrix. The chaotic logistic map is iterated to generate a Latin matrix then randomized to obtain the final S-box. Zhu et al. (2020) proposed a dynamic S-box design method. The final S-box is obtained by applying the fitness function on the proposed static S-box. The static S-box is generated by iterating the logistic-tent system. Solami et al. (2018) proposed an S-box based on the mixing property of a higher dimensional map. A 5-D hyperchaotic system is used to obtain the final S-box. Alhadawi et al. (2021) obtained an S-box utilizing a cuckoo search algorithm and a 1-D discrete space chaotic map. In Jiang & Ding (2021) author generated an 8 × 8 S-box using chaotic bent functions.

The discussed S-boxes achieved strong cryptographic properties that have been analyzed using performance criteria. However, the DP value of these S-boxes is 0.03906, which shows that the maximum DP value is 0.03906. Moreover, several methodologies have been proposed utilizing mathematical transformation of linear fractional transform combined with the symmetric group, elliptic curve, coset diagram, etc., that have a DP value of 0.03906 (Siddiqui, Naseer & Ehatisham-ul-Haq, 2021; Nizam Chew & Ismail, 2020; Beg et al., 2020; Zahid, Arshad & Ahmad, 2019; Farwa, Shah & Idrees, 2016; Hussain et al., 2013a, 2013b; Ahmad et al., 2020; Hayat, Azam & Asif, 2018; Khan, Ahmed & Saleem, 2019; Aboytes-González et al., 2018; Hussain et al., 2018; Siddiqui et al., 2020). However, few methodologies have generated an S-box with a differential probability of 0.156 (Aboytes-González et al., 2018; Siddiqui et al., 2020; Nizam Chew & Ismail, 2020; Ahmad et al., 2020; Cui & Cao, 2007; Tran, Bui & Duong, 2008). Additionally, recent research on the chaos-based S-box (Özkaynak, 2020) shows that an S-box based on the mixing property of chaotic map has high differential uniformity and nonlinearity. It was observed that exiting hybrid S-box methodologies improve nonlinearity property with chaos and optimization or heuristics. However, differential uniformity of these S-boxes is still high. The nonlinearity property is used in the fitness function as an improvement criterion. The heuristics and optimization-based techniques are an added layer on chaotic mapping to achieve highly nonlinear S-boxes. The nonlinearity of an S-box reflects its resistance against linear cryptanalysis. Chaos-based S-box has better LP as compared to algebraic S-boxes. Despite high differential uniformity of chaos-based S-box, DP property cannot be considered an improvement criterion. The DP property is a good criterion for systematic S-box design. The input/output difference information is required to understand the confusion component for systematic design, along with strong diffusion and key mixing components, makes differential attacks like chosen plaintext/ciphertext attacks infeasible. A recent and notable contribution on chaos-based S-box that uses mixing property of chaotic map and DDT within the design loop to improve the DP value is given in Khan et al. (2018), Khan, Jeoti & Manzoor (2012). It is still challenging to improve the DP value of the chaos-based S-box. It is hypothesized that systematic methodologies, designs based on the knowledge of cryptographic attacks and cryptographic properties as a tool within the methodologies for design, are required to generate S-box with a strong structure. For example, chaos-based S-boxes have a higher DP property value than algebraic S-boxes. The observations of this study are as follows:

1. An S-box is a nonlinear component in an encryption algorithm that provides confusion.
2. An S-box Provides uncertainty that obscures the relationship between plaintext and ciphertext, and a strong encryption algorithm makes chosen plaintext/ciphertext attack infeasible.
3. The low DP value of an S-box indicates high dispersion among $\Delta y$.
4. A strong S-box must have an upper bound of cryptographic performance criteria.
5. Chaos-based S-box has poor cryptographic criteria as compared to algebraic S-box.
6. The cryptographic criterion of DP has remained high in chaos-based S-box.
7. Systematic chaos-based S-box with a solid structure and a good understanding of cryptanalytic attack may lead to a strong S-box with improved cryptographic performance criteria, especially differential uniformity.

### Problem statement

An S-box based on mathematical transformations has near-optimal cryptographic performance criteria compared to a chaos-based S-box. However, a chaos-based S-box can have better immunity against various side-channel attacks (Özkaynak, 2020). A chaos-based S-box with comparable performance criteria to an algebraic S-box is still challenging. Further, it was established that mapping techniques (continuum to integer) to produce an S-box structure are more important than the chaotic system properties (Artuğer & Özkaynak, 2020). Therefore, an S-box solid structure with improved performance criteria can be designed with a good understanding of cryptanalytic attacks, such as linear and differential attacks (Kocarev, 2001).

### Contributions

The contribution of this paper is the use of dispersion property as a new tool to design an S-box. This section explains the use of dispersion property within the design loop to achieve results. While, we mainly focused on presenting the research hypothesis, which is later proved in the results section.

An S-box design is critically essential to resist all known attacks, especially differential cryptanalysis. Cryptosystem having S-box with high DP property value may be prone to chosen-plaintext attacks. It uses plaintext ciphertext pairs to mount differential cryptanalysis. The aim is to recover information without the knowledge of the key. With the help of the cryptosystem's S-box, an attacker tabulates pairs ( $\Delta x,\Delta y$) in DDT and finds DP using Eq. (1). An attacker looks for pairs with a maximum count in DDT to measure the differential uniformity of a given S-box. The dispersion property is employed as a tool to design the proposed S-box. The dispersion property is an added layer provided within the design loop.

For a given n bit S-box $S\!: (0,1)^n \mapsto (0,1)^n$, the dispersion property computes all pairs ( $\Delta {S_x},\Delta {S_y}$), where $\Delta {S_x}$ is the input spread and $\Delta {S_y}$ is the output spread. Similar to DDT, these pairs tabulated in a dispersion matrix (DM). The total number of dispersion pairs and pairs which recur in DM are used to measure the normalized dispersion value between 0 and 1. The normalized dispersion is computed as $\zeta = {{2\;*\;\left| {({d_{total}}\left( \pi \right) \;- \;{d_R}\left( \pi \right))} \right|} \over {T(T\ -\ 1)}}$. The ${d_{total}}(\pi )$ is the total pairs count in DM, ${d_R}(\pi )$ is the total recur pairs count in DM, and ‘*T*’ is the total number of S-box positions. The normalized value of ‘0’ stands for no dispersion, and the ‘1’ entails high dispersion among $\Delta {S_y}$. Further, the normalized value close to 1 shows that the input S-box substituted the input sequence with high randomness, which entails efficient decorrelation among the substituted sequence. Hence, S-box exhibits high nonlinearity. The normalized value of 1 requires ${d_R}\left( \pi \right) = 0$. However, this argument requires distinct pairs in DM. The occurrence of recurring pairs is due to the relative positions of elements in the auxiliary table of an S-box. It can be hypothesized that systematic selection and positioning of elements in the S-box may control the ${d_R}\left( \pi \right)$ in DM.

**Figure 1:**
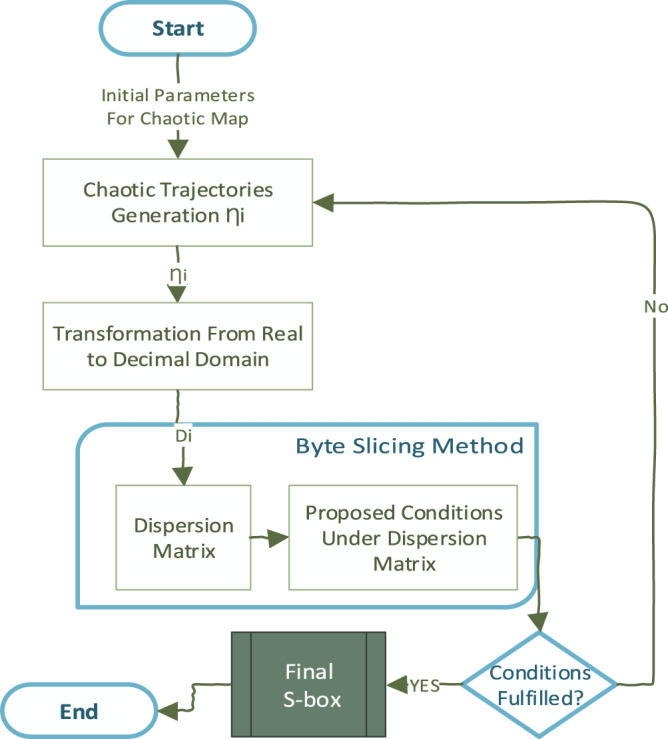
The high-level view of the proposed S-box uses chaos and dispersion.

In the light of the discussion in previous sections, this article attempts to design a systematic S-box using dispersion property within the design loop. The proposed methodology works in layers iteratively. The discretized PWLCM generates initial S-box positions that fill the S-box table. The dispersion property is then used as an added layer that systematically decides the relative position of the S-box element in the S-box table. The proposed method is an increment design approach, starting with an initial pool of S-box positions, using Eq. (3), DM is dynamically generated. The new S-box positions are approved after checking the recurrence of pairs in DM. Due to the dynamic systematic S-box generation, design conditions are proposed under which the DM is dynamically generated, and the relative location of S-box positions are chosen. The recurrence of pairs in DM is closely monitored, and positions are regenerated and placed in the S-box table that entails a high occurrence of pairs in DM. The high-level flow diagram is given in Fig. 1. The ergodic and mixing behavior inherent in chaos generates all S-box positions in a reasonable time. For the added layer of DM generation, each position to confirm as the final S-box position, all pairs are added and checked for recurrence. The time complexity TC of this added layer is closely approximated between ( $O({2^{2n}}) \lt TC \lt O({2^{3n}})$, where *n* is the cardinality of the proposed S-box.

On the other hand, choosing chaos also stands critical in cryptosystem design. In this work, a multi-dimensional PWLCM is chosen to generate initial trajectories. A multi-dimensional map is crucial in resisting key-related attacks in secure chaotic communications systems (Liu, Xiang & Liu, 2020). Initially, a random number generator (RNG) design is proposed using PWLCM. The random numbers generated using PWLCM are cryptographically secure, and statistically analyzed by the National Institute of Standards and Technology (NIST) criterion. This paper is organized as follows: “Random Number Generation Using PWLCM” performs a randomness test of PWLCM, “Materials and Methods” and “Dispersion Matrix Generation” presents the proposed methodology. With the understanding of DDT for differential cryptanalysis that finds the weaknesses in the S-box structure, this research proposes a systematic S-box design. “Proposed Systematic S-box Application in Image Encryption” evaluates the performance of proposed S-boxes. “Boomerang Connectivity Table” and “Feistel counterpart of BCT (FBCT)” analyze the BCT and FBCT of proposed S-box. The performance criteria of chaos-based S-box are not optimal compared to algebraic S-boxes. However, S-box differential uniformity certainly improved as compared to recently proposed S-boxes.

### Random number generation using PWLCM

The randomness of PWLCM is evaluated using the NIST-800-22 statistical test suite. The test suite includes 15 different types of tests. Any bitstream must pass all these tests from the random bitstream pool to be accepted as a successful key and used as a secure key in encryption. A length of one million of the bitstream is required for NIST-800-22 statistical tests. The PWLCM equation is defined as:

(2) $$\eqalign{x_{n+1}= \left\{\matrix{\displaystyle{{x_n}\over {p}}, & 0 \leq x_n \lt p \\ \displaystyle\frac{(x_n - p)}{(0.5-p)}, & p \leq x_n \lt 0.5\\ \displaystyle\frac{(1-p-x_n)}{(0.5-p)},& 0.5 \lt x_n \lt 1-p\\ \displaystyle\frac{(1-x_n)}{p},& 1-p \lt x_n \lt 1 \\ } \right.}$$

where, *x*_*o*_ ∈ [0, 1) is the initial value and *p* ∈ (0, 0.5) is the control factor. Any arbitrary chosen initial condition can be used. It is well established that the randomness of the RNG numbers directly affects encryption applications’ security. Hence they have crucial importance. Therefore, a successful bitstream selected as a key for encryption possesses a property that should have a uniform probability distribution of 1′s and 0′s. It means that the number of 1′s and 0′s in the bitstream should be equal or nearly equal. A PWLCM generates floating-point numbers in the given range of [0–1). As a result, by using PWLCM trajectories, we can generate infinite real number values in this range. A suitable threshold value is set on the continuous-valued output of RNG. Therefore, this paper chooses the typical median value of the threshold, *i.e*., $\tau = 0.5$, bearing in mind the output range of RNG values to be [0–1). The steps for generating a random bit stream using the proposed RNG are as follows:

**Step 1**: The initial condition $({x_0} = 0.78)$ and parameter $(p = 0.16)$ are provided as input to PWLCM for generating random floating-point numbers having a range $[0,1)$.
**Step 2**: The PWLCM is iterated ${10^6}$ times to generate 1 million random floating-point values.
**Step 3**: Thresholding is applied to the floating-point values obtained after step 2 to generate a random bit stream of 0′s and 1′s. Each floating-point value ${x_i}$ (where $1 \le i \le {10^6}$) is mapped to either ‘0’ or ‘1’ depending upon the following criteria: If ${x_i} \ge \tau$, it is mapped to a bit ‘1’; otherwise, the value is mapped to a bit ‘0’. In this way, a bitstream of a length of 1 million is generated using the proposed RNG.
**Step 4**: In the last phase, NIST tests are applied to the bitstream obtained in step 3 to assess the bitstream’s randomness. The test results are evaluated based on a calculated test statistic value, *i.e*., *P*-value, which is a function of the data. The *P*-value reveals the strength of the randomness of a bit sequence. A *P*-value of 1 means the sequence is entirely random, whereas a *P*-value of 0 indicates entirely non-random. For each test, if *P*-value obtained is greater than or equal to the significance level ‘α,’ the test is considered successful. The significance level lies in the range [0.001–0.01]. We used the default parameters for all tests to test our proposed RNG using the NIST test. The value of α was chosen equal to 0.01, which means that for a test to be successful, the *P*-value obtained must be greater than or equal to 0.01. The random bit stream obtained from the proposed RNG using PWLCM passed all NIST tests presented in Table 1.

**Table 1:** Chaotic PWLCM NIST RNG test.

Index	NIST statistical test	*P*-value	Status
1	Frequency (mono bit) test	0.315379	Passed
2	Block Frequency	0.186620	Passed
3	Cumulative Sum test	0.425888 (Forward) 0.202842 (Reverse)	Passed
4	Runs test	0.605161	Passed
5	Longest Run test	0.954527	Passed
6	Rank test	0.287656	Passed
7	Discrete Fourier Transform test	0.679644	Passed
8	Non-overlapping Template Matching test	0.509078	Passed
9	Overlapping Template Matching test	0.045839	Passed
10	Universal Statistical test	0.296564	Passed
11	Approximate Entropy test	0.993287	Passed
12	Random Excursions test	0.582411	Passed
13	Random Excursions Variant test	0.718984	Passed
14	Serial test	0.783850	Passed
15	Linear Complexity test	0.697704	Passed

## Materials and Methods

### Dispersion matrix generation

Generally, the dispersion property is a post-processing technique used to measure the randomness in a sequence. The proposed novel methodology uses dispersion property within the design loop for systematic S-box design. The dispersion property can be defined as:

**Definition 2:***The dispersion measures the irregularity in output spread* $\Delta {{\rm{S}}_{\rm{y}}}$*for a given input spread* $\Delta {{{S}}_{{x}}}$. *For a given substitution π, the list of dispersion pairs of π is defined as*

(3) $${d_{total}}\left( \pi \right) = \{(\Delta {S_x} = \left( {{S_{x + c}} - {S_x}} \right),\Delta {S_y} = \left( {\pi \left( {{S_{y + c}}} \right) - \pi \left( {{S_y}} \right)} \right)\}$$

where $\Delta {S_x}$, and $\Delta {{{S}}_{{y}}}$ is the input and output spread. As described in the contribution subsection, Counting ${d_{total}}(\pi )$ and ${d_R}(\pi )$ computes normalized dispersion.

**Figure 2:**
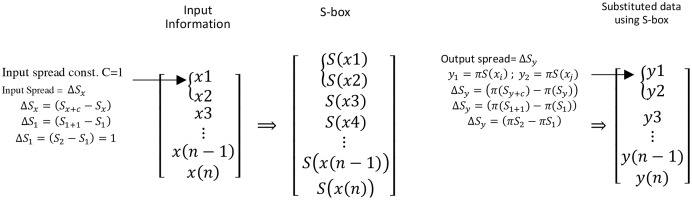
Input and output spread measure using input and substituted data information.

The utilization of dispersion property within the design loop under proposed design conditions requires an understanding of the computation of DM. Figure 2 shows a three-column vector of input information, the S-box, which is used to substitute input information and substituted input information using the S-box, respectively.

The dispersion matrix is filled with the spread pair ( $\Delta {S_x},\Delta {S_y})$. The input spread is measured using input differential with spread variable $C \in (0,255)$. Figure 2 shows the process of measuring $\Delta {S_x}$ and $\Delta {S_y}$. Further, Table 2 demonstrates the process of selecting input spread using the input spread variable to measure the $\Delta {S_y}$. In Table 2, the C = 0 column entails 0, hence not considered herein. Finally, Table 3 shows the dispersion matrix.

**Table 2:** Selection of input differentials using input spread variable C.

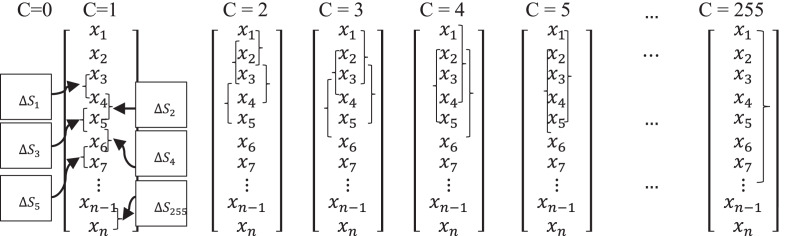

**Table 3:** Generating dispersion matrix by placing forgiven C.

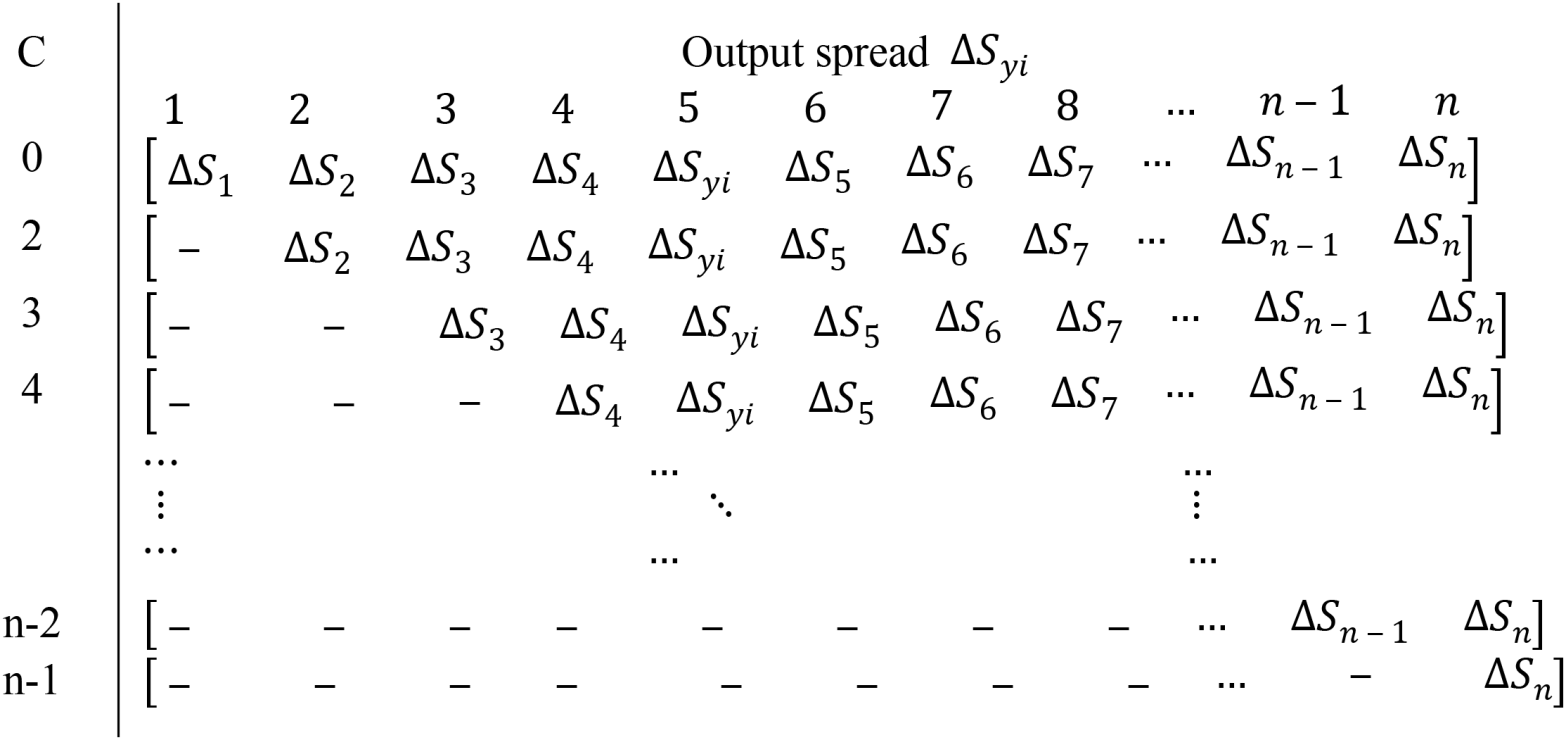

The DM is quite straightforward compared to DDT, which requires a complete S-box for DDT generation. It is further hypothesized that improving the recurrence of pairs in DM may improve the count of output difference in DDT. Therefore the proposed method systematically substitutes S-box elements with low DP value, which seems impossible using a typical chaos-based algorithm (Özkaynak, 2020).

### Steps to design proposed S-box

**1. Variable initialization:**

The first step is to initialize variables used during the proposed design, such as an initial condition for the map ${x_n}$, the final position of the map ${x_{n + 1}}$, position vector PV to store the final S-box.

**2. S-box position mapping:**

The behavior of any generated chaotic trajectories is vetted using the Lyapunov exponent. The nonlinear behavior of the chaotic map to the decimal domain is preserved. The domain in the range [0.1–0.9] is divided into 256 equal intervals, and the intervals are sequentially labeled as position counter PC. In doing so, the generated S-box positions acquire the nonlinear behavior of chaotic trajectories.

**3. Chaotic trajectories decimal mapping:**

The PWLCM is iterated using an arbitrarily chosen initial seed ${x_n}$; however, we use ${x_n} = 0.346$ to generate the proposed S-box, which entails ${x_{n + 1}}$, is checked in the range [0.1–0.9] where it falls and marks associated interval/subdomain number if empty using PC. This PC is an S-box element and stored in a position vector. The S-box’s bijective property is assured by ignoring output value that falls visited subdomain whose PC is already stored in PV ensures distinct positions generation. The chaotic decimal mapping entails an initial S-box.

**4. Systematic byte substitution using dispersion matrix:**

**Figure 3:**
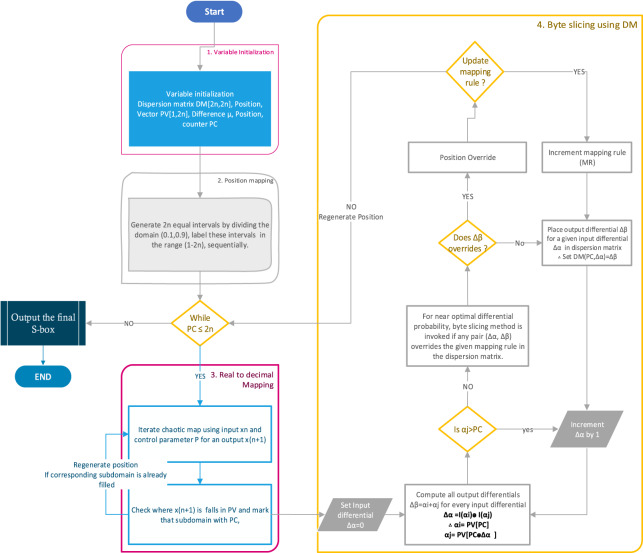
Flow chart of the proposed S-box.

This step ensures the substitution of weak S-box positions that affect the performance parameters of the final S-box. The inherent structure of chaotic trajectories habitually includes these wrong positions as a part of the S-box. Therefore, this work proposed a dispersion matrix-based systematic byte substitution method to generate a near-optimal S-box. The flow graph is presented in Fig. 3. The dispersion matrix is generated within the loop of the proposed S-box design by tabulating the output differential $\Delta {S_y}$ = PV [PC]th and PV[PC - ∆x[i]]th in the dispersion matrix. The dispersion matrix is filled column-wise due to the S-box design’s dynamic nature until each row has a distinct output differential. “Dispersion Matrix Generation” of the proposed methodology details the generation of the dispersion matrix.

1. If the output difference is repeated in any column of the dispersion matrix, the S-box’s corresponding position is ignored and regenerated.
2. Tabulate all output differences of the S-box in the dispersion matrix for all given input differences.
3. The regeneration of S-box positions due to repeated output differences is attempted in the arbitrary given time; otherwise, allow repetition to generate S-box in a reasonable amount of time.
4. Stop iteration once all S-box positions are generated. The position matrix is now the final proposed S-box.

## Results

This section evaluates the proposed systematic S-box’s cryptographic properties, and the results are presented in detail. The performance of the proposed S-boxes is tested and evaluated based on the following parameters: bijection, nonlinearity (NL) (Meier & Staffelbach, 1990), strict avalanche criterion (SAC) (Webster & Tavares, 1986), bit independence criterion (BIC) (Webster & Tavares, 1986; Farwa, Shah & Idrees, 2016), and maximum expected linear and differential probability (Heys, 2002; Matsui, 1996; Hong et al., 2000) and boomerang differential probability (Wagner, 1999; Cid et al., 2018). Numerous researchers have presented tools for verifying an S-box (Wang et al., 2009; Özkaynak, 2019; Picek et al., 2014). The numerical results obtained corresponding to the proposed S-box given in Table 4, are presented in results and discussion sections, verified using the S-box tool. Furthermore, these results are compared with the existing chaos-based S-boxes, algebraic S-box, and other recently proposed methodologies. The following sections briefly explain the S-box testing parameters and discuss their numerical results obtained for the proposed S-box.

**Table 4:** Proposed improved S-box.

Index	0	1	2	3	4	5	6	7	8	9	a	b	c	d	e	f
0	124	26	158	11	115	3	92	31	169	46	204	218	243	126	47	212
1	200	224	174	65	239	25	5	97	15	128	82	69	245	105	42	235
2	147	35	178	81	71	12	118	191	240	122	85	56	222	62	99	10
3	114	8	166	34	48	238	139	206	214	148	181	23	33	4	209	132
4	72	249	108	201	176	13	250	160	95	21	195	59	220	185	86	123
5	36	165	171	1	196	154	73	27	54	84	64	221	197	30	53	217
6	137	141	254	207	203	103	237	140	236	43	231	230	161	184	104	75
7	253	20	6	2	193	120	14	19	80	7	110	74	129	117	24	102
8	93	28	163	219	187	246	116	143	252	70	119	40	241	130	91	183
9	127	49	175	244	98	125	211	190	76	37	66	173	228	164	29	0
a	189	179	83	146	96	18	155	87	113	133	38	112	106	135	90	159
b	100	153	255	247	213	182	107	89	234	150	52	215	232	194	68	208
c	202	22	32	172	223	177	186	168	198	229	162	58	227	192	109	251
d	101	16	136	210	44	225	9	111	151	226	149	41	138	180	17	60
e	134	63	61	77	45	88	79	78	199	167	57	242	121	216	144	156
f	145	170	94	50	142	67	233	188	39	131	157	152	248	55	205	51

**1. Bijective**

The bijection test evaluates the uniqueness of the output of an S-box. If an S-box fulfills the bijection criterion, its output values are unique and non-repeating in the interval $[1,{2^{n - 1}}]$. Also, there is a one-to-one mapping between each input and output value. It can be observed that the proposed S-box satisfies the bijection test. Each S-box produces unique output values in the interval [0, 255], and there is a one-to-one mapping between every input and output.

**2. Nonlinearity**

The nonlinearity (NL) test measures the smallest Hamming distance of the reference function from all the affine functions (Meier & Staffelbach, 1990; Webster & Tavares, 1986; Farwa, Shah & Idrees, 2016). It represents the number of bits that must be altered in the truth table of a Boolean function to approach the nearest affine function. Mathematically, the nonlinearity of a Boolean function is defined as follows:

(4) $$N_f = 2^{n-1} (1-2^{-n}\max | S_{(g)}(W)|)$$

where ${S_{\left( g \right)}}(W)$ represents the Walsh spectrum, which is defined as:

(5) $${S_{\left( g \right)}}\left( w \right) = \sum\nolimits_{w \in GF\left( {{2^n}} \right)} {{{\left( { - 1} \right)}^{g\left( x \right) \oplus x.w}}}$$

The maximum possible nonlinearity value in $GF({2^n})$ is $N = {2^n} - {2^{{n \over 2} - 1}}$ (Nyberg, 1991). Hence, the maximum achievable nonlinearity is 120. The values of nonlinearity achieved for the proposed S-boxes with different initial conditions are given in Table 5. Our proposed S-box provides a minimum and maximum nonlinearity of 100 and 108, respectively. The average nonlinearity achieved with the proposed S-box is between 103.5 and 105.5, which falls under good nonlinearity.

**Table 5:** Nonlinearities of proposed S-boxes.

Index	Initial condition of proposed S-box	f0	f1	f2	f3	f4	f5	f6	f7	Avg. nonlinearity
1	0.165	104	102	100	106	100	106	106	106	103.75
2	0.266	100	106	102	104	106	104	100	106	103.5
3	0.281	104	106	104	106	106	102	106	102	104.5
4	0.341	102	106	108	104	100	100	102	108	103.75
5	0.467	104	100	106	108	100	106	104	102	103.75
6	0.529	102	104	108	104	106	106	106	108	105.5
7	0.632	102	102	100	104	104	106	106	110	104.25
8	0.664	102	104	104	106	100	104	106	100	103.25
9	0.771	108	106	106	104	102	106	102	104	104.75
10	0.863	104	100	104	102	100	104	106	108	103.5
11	0.849	108	104	104	110	102	100	102	104	104.25

**3. Strict avalanche criterion**

Strict Avalanche Criterion (SAC) (Webster & Tavares, 1986) measures how many output bits change for a function when a single input bit is altered. If a function satisfies the SAC, each output bit should change with a probability of one-half whenever a single input bit is complemented. In other words, changing a single input bit should change almost one-half of the output bits. For an S-box to be ideal, the SAC value should be equal to 0.5. The proposed S-box generated SAC and SAC offset values with the proposed scheme (Table 6) achieves an average SAC value approximately equal to 0.5. Additionally, the SAC values obtained are comparable to the existing S-boxes, which shows that the proposed S-boxes satisfy the SAC test.

**Table 6:** SAC values of proposed S-box.

Index	1	2	3	4	5	6	7	8
1	0.546	0.484	0.5	0.484	0.484	0.484	0.562	0.437
2	0.546	0.453	0.531	0.406	0.515	0.484	0.5	0.578
3	0.515	0.406	0.531	0.593	0.468	0.5	0.531	0.531
4	0.5	0.484	0.468	0.468	0.531	0.437	0.437	0.437
5	0.390	0.453	0.5	0.453	0.453	0.484	0.515	0.515
6	0.468	0.515	0.437	0.562	0.5	0.562	0.484	0.546
7	0.406	0.593	0.484	0.562	0.468	0.484	0.546	0.421
8	0.531	0.531	0.5	0.546	0.484	0.484	0.515	0.531

**4. Bit independence criteria**

The output bit independence criterion (BIC) is a crucial property for any cryptographic system and was introduced by Webster & Tavares (1986) to analyze the behavior of bit patterns at the output. A single plaintext bit is altered for investigating the BIC, and the output binary vectors are analyzed for independence. All avalanche variables must be pair-wise independent for a given set of avalanche vectors generated by complementing a single plaintext bit to satisfy the BIC. The correlation between an input-output pair measures the amount of independence among all avalanche pairs. For two variables, A and B, correlation presented in mathematical form as follows:

(6) $$\rho \left\{ {A,B} \right\} = {{{\rm{cov}}\left\{ {A,B} \right\}} \over {\sigma \left\{ A \right\}\sigma \left\{ B \right\}}}$$

where, $p\{ A,B\}$, and $cov\{ A,B\}$ is the correlation coefficient and covariance of $A$ and $B$, respectively. The proposed S-boxes achieve an average BIC value of 108 each, equal or better than most of the existing S-boxes is given in Tables 7 and 8. Thus the proposed S-box successfully fulfills the BIC.

**Table 7:** SAC values for BIC of proposed S-box.

Index	1	2	3	4	5	6	7	8
1	–	0.482	0.492	0.516	0.490	0.504	0.482	0.477
2	0.482	–	0.486	0.498	0.512	0.518	0.510	0.494
3	0.492	0.486	–	0.510	0.508	0.490	0.492	0.520
4	0.516	0.498	0.510	–	0.508	0.508	0.506	0.492
5	0.490	0.512	0.508	0.508	–	0.475	0.480	0.490
6	0.504	0.518	0.490	0.508	0.475	–	0.525	0.498
7	0.482	0.510	0.492	0.506	0.480	0.525	–	0.494
8	0.477	0.494	0.520	0.492	0.490	0.498	0.494	–

**Table 8:** BIC values for the nonlinearity of the proposed S-box.

Index	1	2	3	4	5	6	7	8
1	–	106	106	106	100	108	106	106
2	106	–	100	102	106	100	106	98
3	106	100	–	108	106	106	106	104
4	106	102	108	–	106	100	100	106
5	100	106	106	106	–	104	110	106
6	108	100	106	100	104	–	104	106
7	106	106	106	100	110	104	–	108
8	106	98	104	106	106	106	108	–

**5. Linear approximation probability**

**Figure 4:**
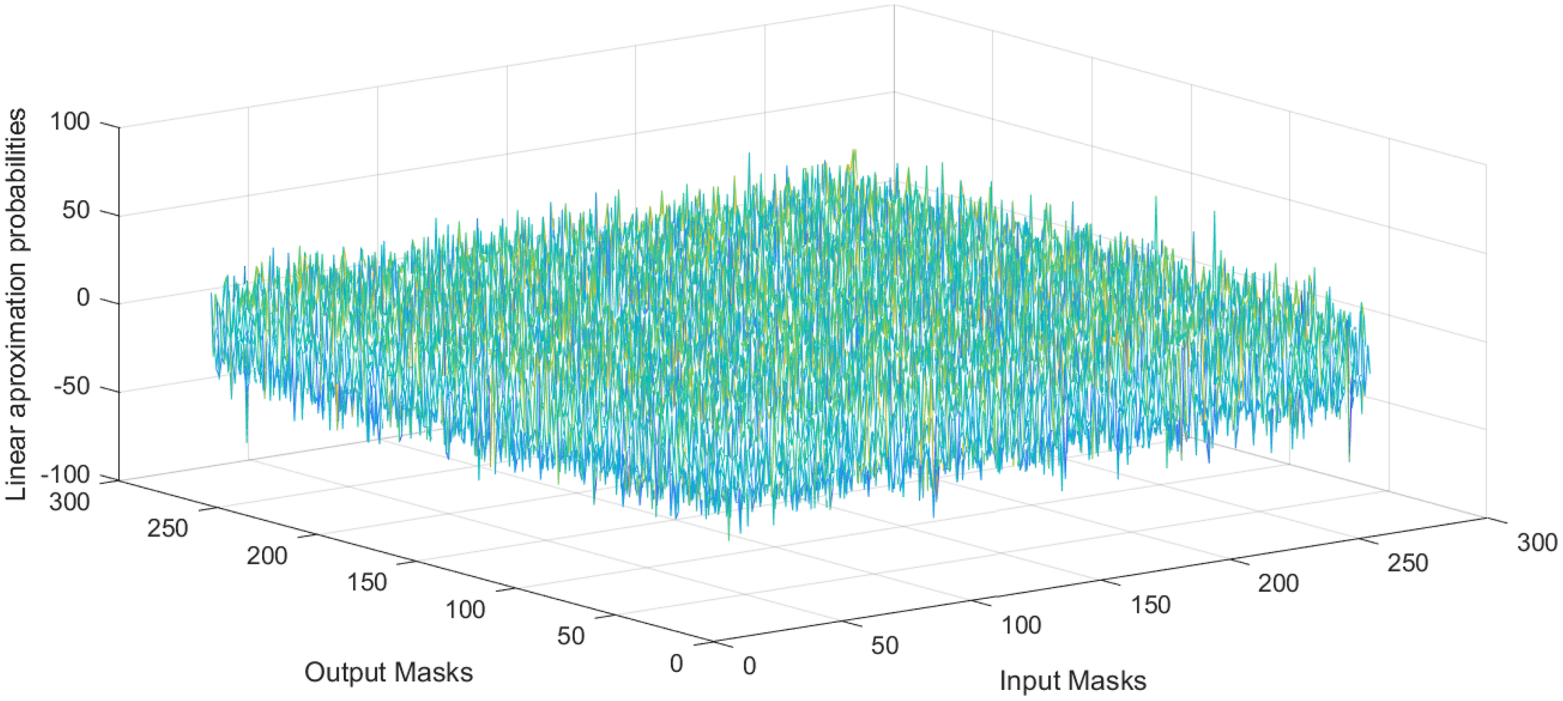
Histogram of LAT of proposed S-box.

The linear approximation probability (LP) measures the maximum imbalance between input and output bits (Aboytes-González et al., 2018). Mathematically, the linear approximation probability of an S-box is defined as:

(7) $$LP = {\max _{{\Gamma _x},{\Gamma _y} \ne 0}}\left| {{{\# \left\{ {x|x.{\Gamma _x} = S\left( x \right).{\Gamma _y}} \right\}} \over {{2^n}}} - {1 \over 2}} \right|$$

where ${\Gamma _x}$ and ${\Gamma _y}$ are input and output masks, respectively, x is the set of all possible input values, and ${2^n}$ is the number of S-box elements. The LP value of the proposed S-box is 0.1028, which show that the proposed S-boxes achieve efficient performance in term of the proposed S-box’s LP. The linear approximation table S-box is given in Table 9. Further, the histogram of LAT of the proposed S-box is given in Fig. 4. As a result, the S-boxes generated using the proposed method are resilient to linear cryptanalysis.

**Table 9:** LAT of proposed S-box.

	0	1	2	3	4	5	6	7	8	9	a	b	c	d	e	f
0	128	0	0	0	0	0	0	0	0	0	0	0	0	0	0	0
1	0	4	−12	8	8	4	4	−16	0	4	−4	0	−16	12	−12	−16
2	0	−8	−28	4	8	0	−12	4	−32	24	4	−12	8	16	4	−12
3	0	12	0	−28	−8	−4	−8	36	16	−4	−8	28	−16	4	−24	4
4	0	−16	4	20	12	−28	8	0	−32	−8	28	4	4	−12	−8	−8
5	0	−20	16	−20	36	16	−28	0	8	−4	24	−4	−20	−16	12	0
6	0	16	0	−24	20	12	4	4	24	−16	−16	0	20	20	−20	−44
7	0	−4	−12	24	−12	−16	0	20	16	−12	36	0	20	24	−16	28
8	0	8	8	0	−12	20	−4	−4	28	−20	4	4	−48	8	8	0
9	0	−12	12	−16	12	0	16	−28	12	−24	−16	−20	32	−20	12	8
a	0	8	−20	−4	−12	−28	24	−16	4	−12	16	−8	8	32	−20	−4
b	0	4	−8	4	20	−24	12	8	−28	−16	−12	8	−16	−20	32	36
c	0	−8	−20	4	−24	16	12	4	4	−4	−8	0	−12	28	16	24
d	0	−4	−8	20	0	−12	−8	−36	12	40	4	0	−4	48	4	8
e	0	0	−24	16	−8	−8	0	8	20	20	4	−20	20	4	36	−4
f	0	20	−4	24	−8	20	44	16	−20	0	8	20	20	0	8	12

**6. Differential approximation probability**

The differential approximation probability (DP) exhibits the differential uniformity of an S-box (Aboytes-González et al., 2018; Hong et al., 2000), which is mathematically defined as given in (8).

(8) $$DP\left( {\Delta x \to \Delta y} \right) = \left( {{{\# \left\{ {x \in X|S\left( x \right) \oplus S\left( {x \oplus \Delta x} \right) = \Delta y} \right\}} \over {{2^n}}}} \right)$$

**Figure 5:**
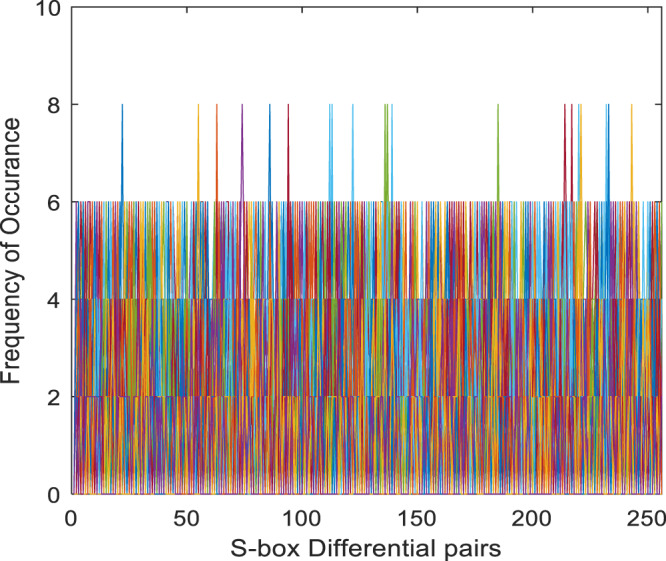
Frequency of occurrence of DDT elements of proposed S-box.

**Figure 6:**
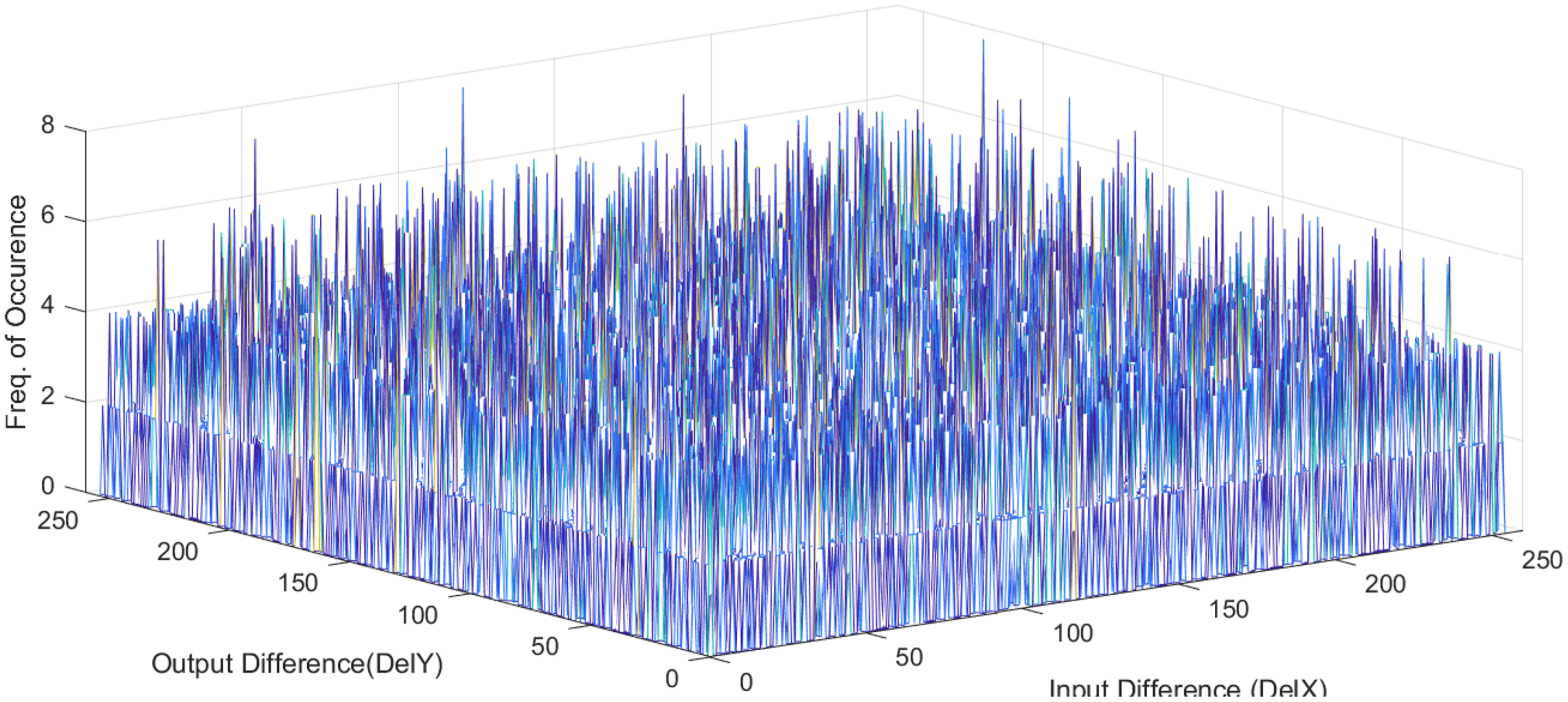
Histogram of DDT of proposed S-box.

$\Delta x$ and $\Delta y$ are input and output differential, respectively, ${\rm{X}}$ is the set of possible input values, and ${2^n}$ is the number of S-box elements. An S-box with lower differential uniformity is considered cryptographically secure. This research aims to propose a systematic S-box methodology to improve the differential uniformity. The DDT of the proposed S-box is shown in Table 10. The proposed S-box has a differential uniformity of 8 and a maximum DP value of 0.03125. The obtained maximum DP value of the proposed S-box is compared with existing related S-box methodologies and tabulated in Table S2. For chaos-based S-box, the maximum DP value of 0.03125 is considered near-optimal compared to most existing S-boxes. The frequency of occurrence of $\Delta y$ in DDT is shown in Fig. 5. Further, the histogram of DDT of the proposed S-box is given in Fig. 6. It shows that the proposed S-box improves the occurrence of $\Delta y$ in DDT and 98% of the $\Delta y$ occurs with the probability of 0.234. Hence, we prove the hypothesis that controlling the ${d_{R(\pi )}}$ under given design conditions by systematically chosen S-box position entails improved occurrence of $\Delta y$ in DDT. Therefore, it is concluded that the proposed scheme ably generates S-boxes that are core security components in encryption algorithms and provide strong security to resist cryptanalytic attacks.

**Table 10:** DDT of proposed S-box.

Index	0	1	2	3	4	5	6	7	8	9	a	b	c	d	e	f
0	0	6	6	6	6	6	6	8	6	6	4	6	6	6	6	6
1	6	6	6	6	6	6	6	6	6	4	6	6	4	6	4	6
2	6	6	6	4	6	6	6	6	6	6	6	6	6	6	6	8
3	6	6	6	6	6	6	6	6	6	6	6	6	6	6	6	6
4	6	6	6	6	6	4	6	6	6	6	6	6	6	6	6	6
5	6	8	6	6	6	8	6	6	6	6	6	6	6	8	6	6
6	6	6	6	8	6	6	6	6	6	4	6	6	6	6	6	4
7	6	6	6	6	6	4	6	6	8	6	6	6	6	6	8	6
8	6	6	6	6	6	6	6	6	8	4	6	8	6	8	8	4
9	6	6	6	6	8	6	6	8	6	6	6	6	6	6	6	6
a	6	6	6	6	6	6	6	6	8	6	6	6	6	6	6	6
b	6	6	6	4	6	6	6	6	6	6	4	6	4	8	6	6
c	6	6	4	4	6	6	6	6	6	6	6	6	6	8	6	6
d	6	6	6	6	6	8	6	6	6	6	6	6	6	4	6	6
e	6	6	6	8	6	6	6	6	6	6	6	6	6	6	6	6
f	6	6	6	6	6	6	8	6	6	6	6	6	6	6	6	6

**7. Correlation analysis: sensitivity among S-boxes**

**Figure 7:**
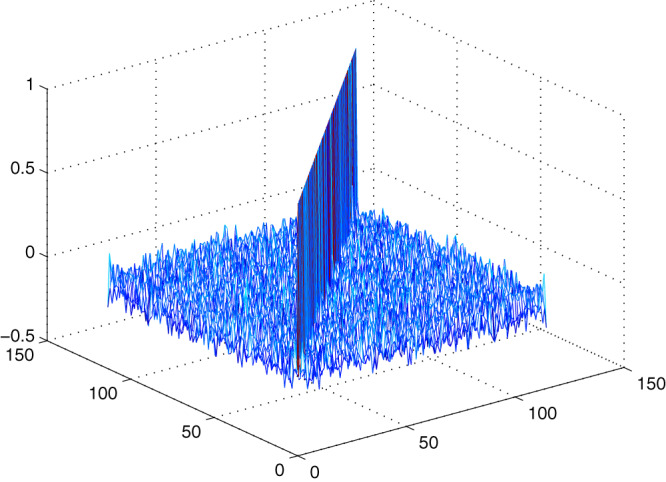
Correlation among proposed S-boxes with a slight change in IC.

**Figure 8:**
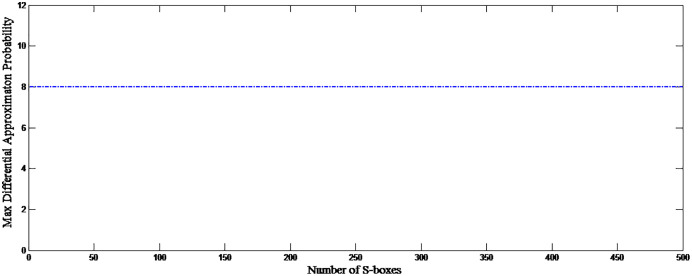
Differential uniformity of proposed S-boxes.

To study the randomness among S-boxes, the correlation coefficient is measured. It determines the similarities among S-boxes with a slight change in the initial condition. The correlation coefficient, ρ, is measured as:

(9) $$cov\left( {{S_i},{S_j}} \right) = {1 \over N}\sum\limits_{k = 1}^N {\left( {{S_i}\left( k \right) - E\left( {{S_i}} \right)} \right)} \left( {{S_j}\left( k \right) - E\left( {{S_j}} \right)} \right)$$

(10) $$\rho \left( {{S_i},{S_j}} \right) = {{cov\left( {{S_i},{S_j}} \right)} \over {\sigma \left( {{S_i}} \right)\sigma \left( {{S_j}} \right)}}$$

where $E(S_i) = \frac{1}{N}\sum_{k=1}^{N}S_k, \sigma (S_i) = \sum_{k=1}^{N} (S_k - ES_k)^2$, and ${N = 2^n}$ where *n* = size of the S-box. The initial condition is changed to the 4th decimal digit for the analysis, and 500 S-boxes are generated. Figure 7 shows the correlation among proposed S-boxes. The *x*-axis shows the number of inputs, the *y*-axis shows the number of S-boxes, and the *z*-axis shows the values of correlation coefficients. The upper and lower bound of the achieved correlation coefficient ranges from −0.2139 to 0.2667. It is quite evident from the Fig. 7 that the proposed S-boxes have very low correlation coefficient values. The correlation of the S-box gives the value of 1, as shown with a diagonal bar in Fig. 7. The differential uniformity of all generated S-boxes is measured and plotted in Fig. 8. Hence, it proves the hypothesis that it retains good DP values and inherent design technique results in highly uncorrelated S-boxes. Therefore, the proposed S-box method is highly suitable to design key-based S-boxes.

**8. Boomerang connectivity table (BCT)**

**Figure 9:**
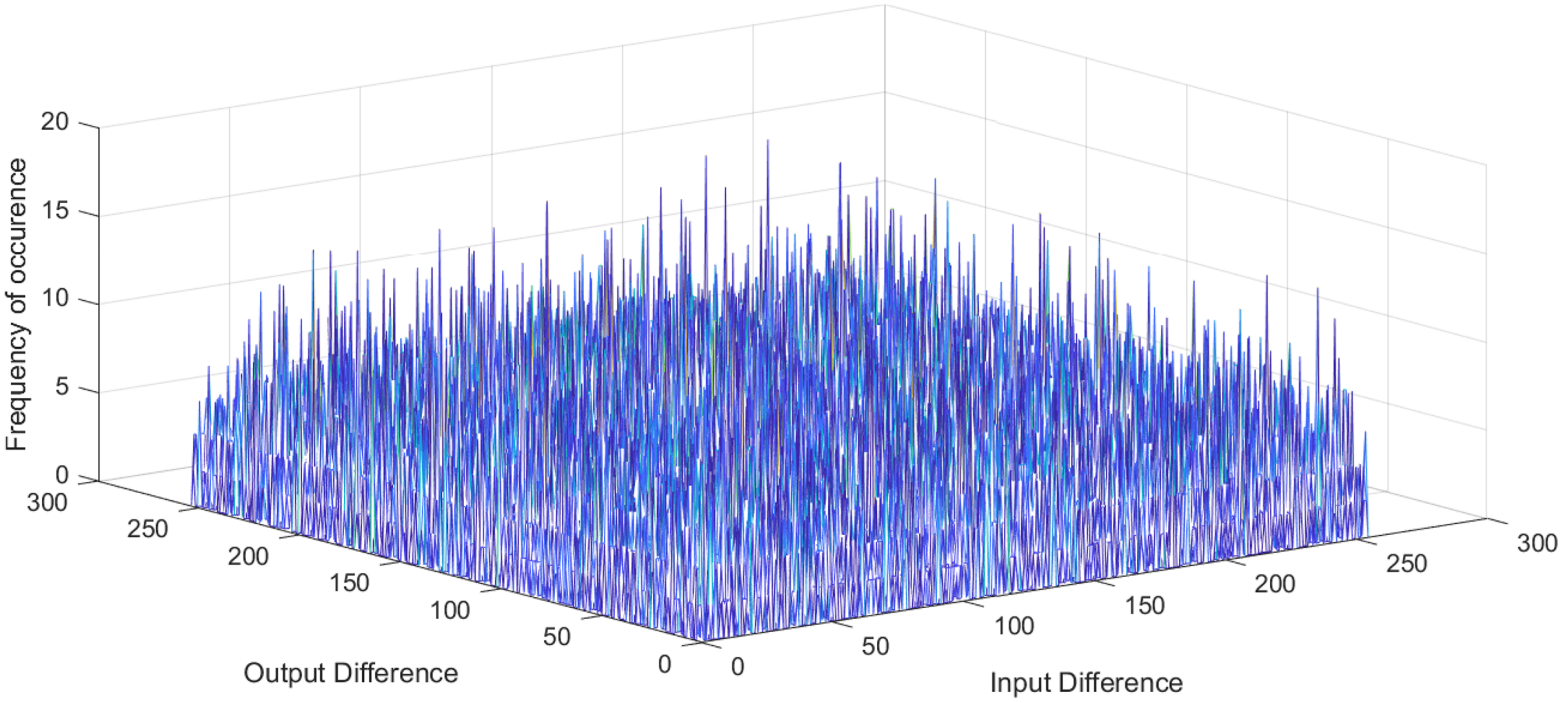
Histogram of BCT of proposed S-box.

The boomerang attack, proposed by Wagner (1999), is a popular cryptanalytic technique used to analyze the security of a block cipher. The boomerang connectivity table (BCT), proposed by Cid et al. (2018), is an efficient and simple method that accurately measures the connection probability for a boomerang-styled attack. Like the DDT, BCT provides useful information for analyzing an S-box for a cryptosystem. Therefore, the strength of an S-box as a confusion component can be measured using BCT. For a given input difference ${\Delta _i}$, the BCT computes the probability of boomerang of ${\Delta _i}$ using output difference ${\nabla _o}$ for all values of input x. The BCT computes all pairs ( ${\Delta _i},{\nabla _o}$) using the following equation,

(11) $$\# \left\{ {x \in {{\left\{ {0,1} \right\}}^n}|{S^{ - 1}}\left( {S\left( x \right) \oplus {\nabla _o}} \right) \oplus {S^{ - 1}}\left( {S\left( {x \oplus {\Delta _i}} \right) \oplus {\nabla _o}} \right) = {\Delta _i}} \right\}$$

where ${S^{ - 1}}$ is the inverse of an S-box, ${\Delta _i}$ and ${\nabla _o}$ is the input and output difference, respectively. The BCT, given $({\Delta _i},{\nabla _o})$ and for all input x, determine and tabulate in BCT, the probability of boomerang of ${\Delta _i}$. There is a deep relationship between BCT and DDT (Cid et al., 2018). The number of entries in BCT is greater than or equal to DDT, with the proportion given in Song, Qin & Hu, 2019. The BCT table of the proposed S-box is given in Table 11. The histogram of BCT of size $256 \times 256$ of our proposed S-box is given in Fig. 9.

**Table 11:** BCT table of proposed S-box.

Index	0	1	2	3	4	5	6	7	8	9	a	b	c	d	e	f
0	256	256	256	256	256	256	256	256	256	256	256	256	256	256	256	256
1	256	8	6	8	4	14	4	4	4	12	12	4	4	6	6	2
2	256	6	4	6	8	2	4	6	6	6	6	10	10	8	4	2
3	256	8	2	6	2	2	6	4	12	2	8	2	6	6	4	4
4	256	12	2	6	2	14	4	4	4	2	2	2	4	6	2	4
5	256	12	2	6	2	2	2	2	2	4	6	4	2	2	4	8
6	256	8	6	10	4	8	6	4	2	6	14	4	6	4	2	6
7	256	8	2	10	2	2	14	16	6	12	4	4	2	2	12	4
8	256	8	12	12	2	8	14	6	4	4	8	2	2	2	6	6
9	256	12	10	12	8	2	4	4	2	12	8	4	2	4	4	4
a	256	8	2	4	2	2	14	4	4	2	4	2	2	4	8	10
b	256	8	4	4	4	8	14	4	4	4	14	6	4	2	2	8
c	256	12	4	14	4	4	4	14	4	14	6	4	6	2	2	12
d	256	16	8	4	4	8	2	8	8	4	2	16	2	4	6	6
e	256	12	2	8	6	4	12	4	4	4	2	4	6	2	4	6
f	256	8	2	4	4	4	4	8	8	4	4	16	8	6	8	8

**Figure 10:**
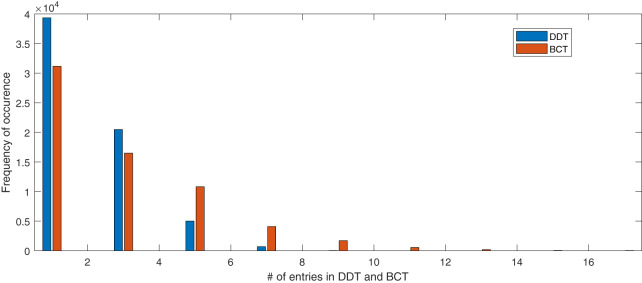
Number of entries in BCT and DDT of proposed S-box.

The entries in the first row and first column of BCT are all 256. For a better illustration of the internal structure of BCT, Fig. 9 does not include the first row and first column of the BCT. The frequency of each entry in BCT and DDT of our proposed S-box is summarized in Table 12. Due to the inherent generation structure of BCT, the differential uniformity in BCT is 16 with 21 entries. In comparison, differential uniformity in DDT is 8 with 20 entries. The number of BCT and DDT entries of proposed S-box can be visualized in Fig. 10.

**Table 12:** The number of entries for each value in DDT and BCT of the proposed S-box and AES S-box.

S-box	Table	256	16	14	12	10	8	6	4	2	0
Proposed	BCT	511	21	48	174	511	1,704	4,072	10,808	16,480	31,166
	DDT	1	–	–	–	–	20	688	5,016	20,464	39,347
AES	BCT	511	–	–	–	–	–	510	255	31,620	32,640
	DDT	1	–	–	–	–	–	–	255	32,130	33,150

A detailed analysis is provided in Cid et al. (2018) for desired BCT differential uniformity of $4 \times 4\ and\ 8 \times 8$ S-boxes to resist boomerang attack and later Boura & Canteaut (2018) provided the best possible differential uniformity of BCT for $4 \times 4$ S-boxes. However, the best possible differential uniformity of BCT for $8 \times 8$ S-box still is an open problem.

**9. Feistel counterpart of BCT (FBCT)**

Another related extension of BCT for ciphers following Feistel construction was proposed by Boukerrou et al. (2020). For a given S-box, the Feistel counterpart BCT (FBCT) is defined as:

(12) $$FBCT\left( {{\Delta _i},{\nabla _o}} \right) = \# \{x \in {\left\{ {0,1} \right\}^n}|S\left( x \right) \oplus S\left( {x \oplus {\Delta _i}} \right) \oplus (S\left( {x \oplus {\Delta _i}} \right) \oplus \left( {x \oplus {\Delta _i} \oplus {\nabla _o}} \right) = 0\}$$

The FBCT was given $\left( {{\Delta _i},{\nabla _o}} \right)$ and for all values of x, the probability that (12) hold is computed and tabulated in FBCT. Some direct properties of FBCT are given as:

(1) Symmetry: for all $0 \le {\Delta _i},{\nabla _o} \le {2^n} - 1$, FBCT(0, ${\nabla _o}$) $= {2^n}$
(2) Fixed value :

 (a) First row: for all $0 \le {\nabla _o} \le {2^n} - 1$, FBCT $({\nabla _o},0) = {2^n}$ (ladder switch)
 (b) First column: for all $0 \le {\Delta _i} \le {2^n} - 1$, FBCT $({\Delta _i},0) = {2^n}$ (ladder switch)
 (c) Diagonal: for all $0 \le {\Delta _i} \le {2^n} - 1$, FBCT $({\Delta _i},{\Delta _i}) = {2^n}$ (Feistel switch)

(3) Multiplicity: for all $0 \le {\Delta _i},{\nabla _o} \le {2^n} - 1$, FBCT $({\Delta _i},{\nabla _o}) \equiv 0\ mod\ 4$
(4) Equalities: for all $0 \le {\Delta _i},{\nabla _o} \le {2^n} - 1$, FBCT $({\Delta _i},{\nabla _o}) = {\rm{FBCT}}({\Delta _i},{\Delta _i} \oplus {\nabla _o})$

**Figure 11:**
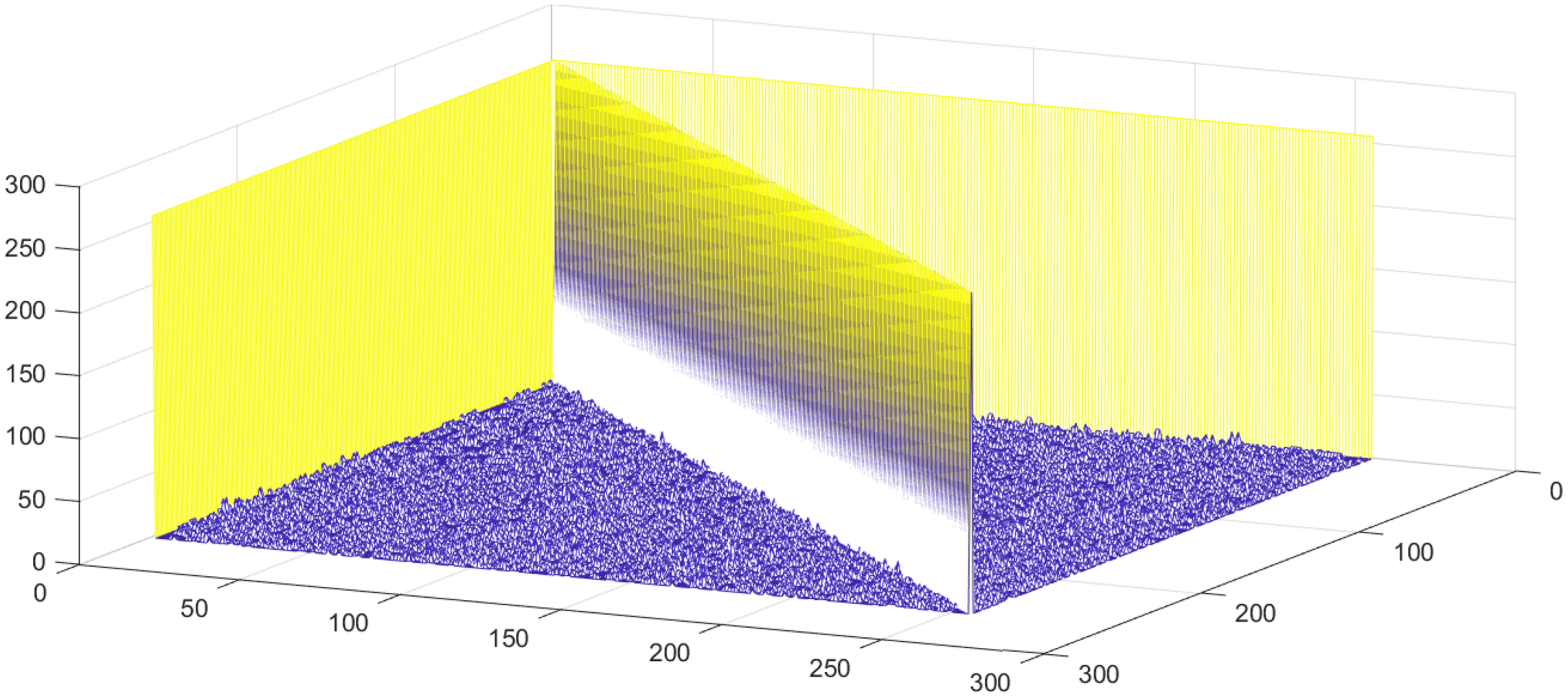
Histogram of FBCT of proposed S-box.

**Figure 12:**
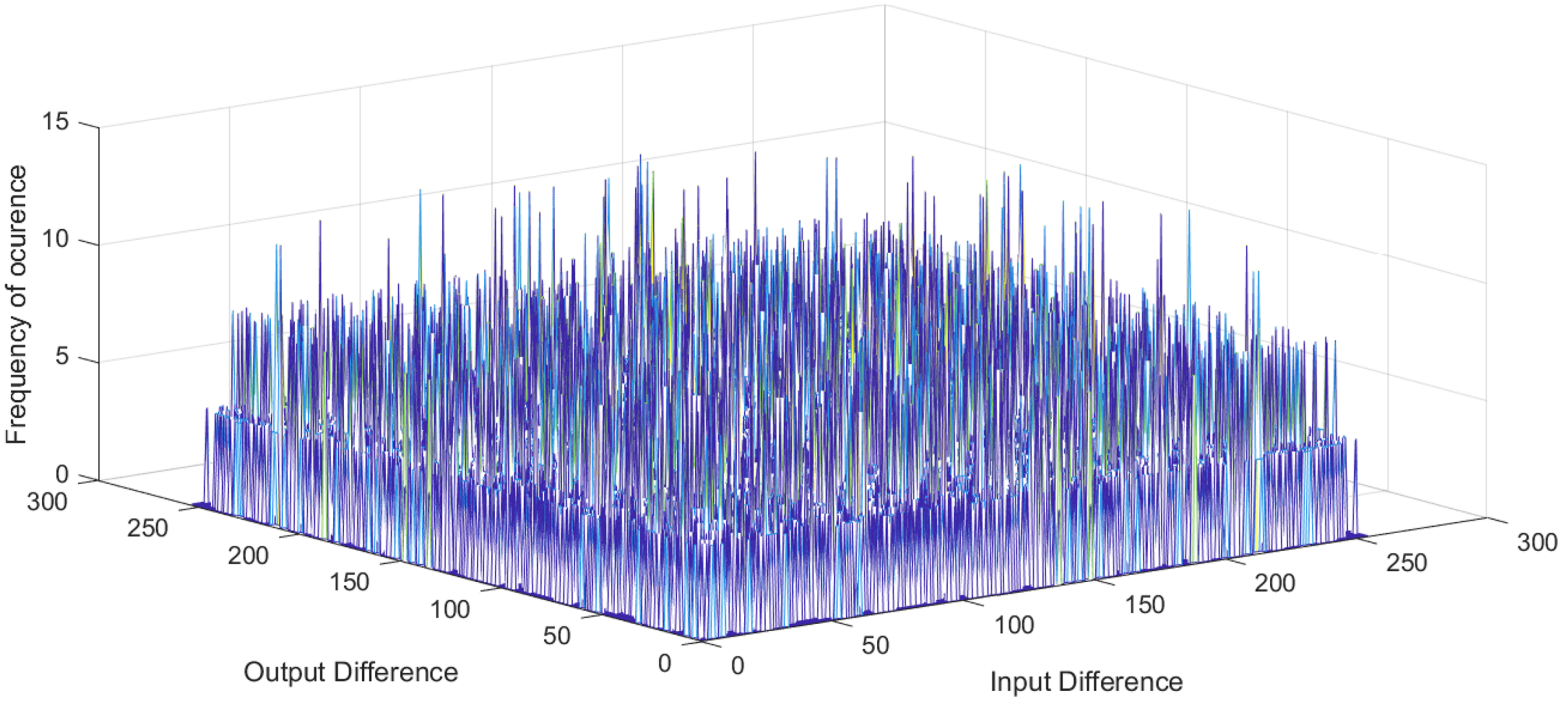
Histogram of FBCT of proposed S-box without considering the entries value.

Boukerrou et al. (2020) addressed the detailed proof of said properties and similarities between BCT and FBCT. The histogram of FBCT of the proposed S-box with and without conserving the entries of ${2^n}$ in FBCT is given in Figs. 11 and 12. The FBCT entry values at the first row, first column, and diagonal is ${2^n}$. The entries of ${2^n}\;{\rm{in}}\;{\rm{diagonal}}$ of FBCT is called the Feistel switch. The F-boomerang uniformity ( ${\beta ^F}$), the highest value in FBCT, ignoring the first row, first column, and diagonal is ${\beta ^F} \ge 4$. The F-boomerang uniformity of the proposed S-box is ${\beta ^F} = 12$. In FBCT, number of entries of each value of 256, 12, 8, 4, and 0 is 766, 51,990, 11,274, 1,392, and 114, respectively.

### Proposed systematic S-box application in image encryption

The suitability of the proposed S-box is evaluated as an application in image encryption. Image encryption, measures the strength and robustness of the proposed S-box, is performed using majority logic criteria (MLC) (Hussain et al., 2012; Shah et al., 2011). It is presented herein just to showcase the capability of the proposed S-box and not being used as a cipher. We used a standard gray-level San Diego aerial image of size 512 $\times$ 512 as plaintext to perform the substitution (Weber, 1981). This image can be used freely for research purpose. This image was substituted using the proposed S-box and AES S-box individually. The S-box substituted the pixel values of an image with the corresponding value in the S-box. The ciphertext is the scrambled image that hides the visual information contained in the plaintext. We performed a single round image substitution to perform some statistical analysis on plain and encrypted images. We performed these statistical analyses, namely histogram analysis, entropy, energy, correlation, contrast, and homogeneity analysis.

It can be observed from Table 13 that the proposed systematic S-box efficiently disperse the correlated pixels that provide effective image substitution. Results show that parameters are mainly comparable to the AES S-box. The entropy parameter value obtained using the proposed systematic S-box is 7.4060, near the superior value of 8. The entropy value indicates the randomness in an image. Hence, the proposed S-box is designed to provide near-optimal decorrelation between input and output elements in the image, amplifying randomness. The energy parameter value of the plain image is 0.0780. When image encryption is applied to plain images, we achieved an energy value of 0.0161, the same as the AES S-box energy value. The achieved energy value is small, which entails efficient image encryption performance of the proposed S-box. The correlation shows the linear independence between plain and encrypted images.

**Table 13:** Statistical analysis of image substitution of proposed S-box.

Statistical analysis	Plain San Diego aerial image	Encrypted San Diego aerial image with AES S-box and proposed S-box	
		AES S-box	Proposed S-box
Entropy	7.4061	7.4060	7.4060
Energy	0.0780	0.0158	0.0161
Correlation	0.7724	0.0155	0.0398
Contrast	1.0969	10.223	9.9895
Homogeneity	0.7255	0.4014	0.4087

The coefficient value of approximately 0 indicates no or weak correlation between images. The proposed S-box’s correlation parameter value is 0.0398, close to 0, and comparable with AES S-box. The proposed S-box enhances the spread and dispersion among input and output pixels. Thus, it results in a weak correlation among pixels values. Further, the proposed S-box enhances the modern encryption properties of confusion and diffusion. The contrast parameter value of the proposed S-box is 9.9895. The constant image entails a contrast value of 0. A high value of contrast indicates randomness in the image. Due to systematic nonlinear mapping using the proposed S-box, Objects in the plain image are dispersed completely. Therefore, we achieved a high value of contrast in encrypted that indicates strong encryption. The homogeneity parameter measures the closeness of the distributed pixels of GLCM to its diagonals. The achieved homogeneity results using the proposed S-box and AES S-box are comparable and show strong encryption. Using majority logic, the image substitution analysis entails the proposed S-box results comparable to the state-of-the-art results in Table 13.

**Figure 13:**
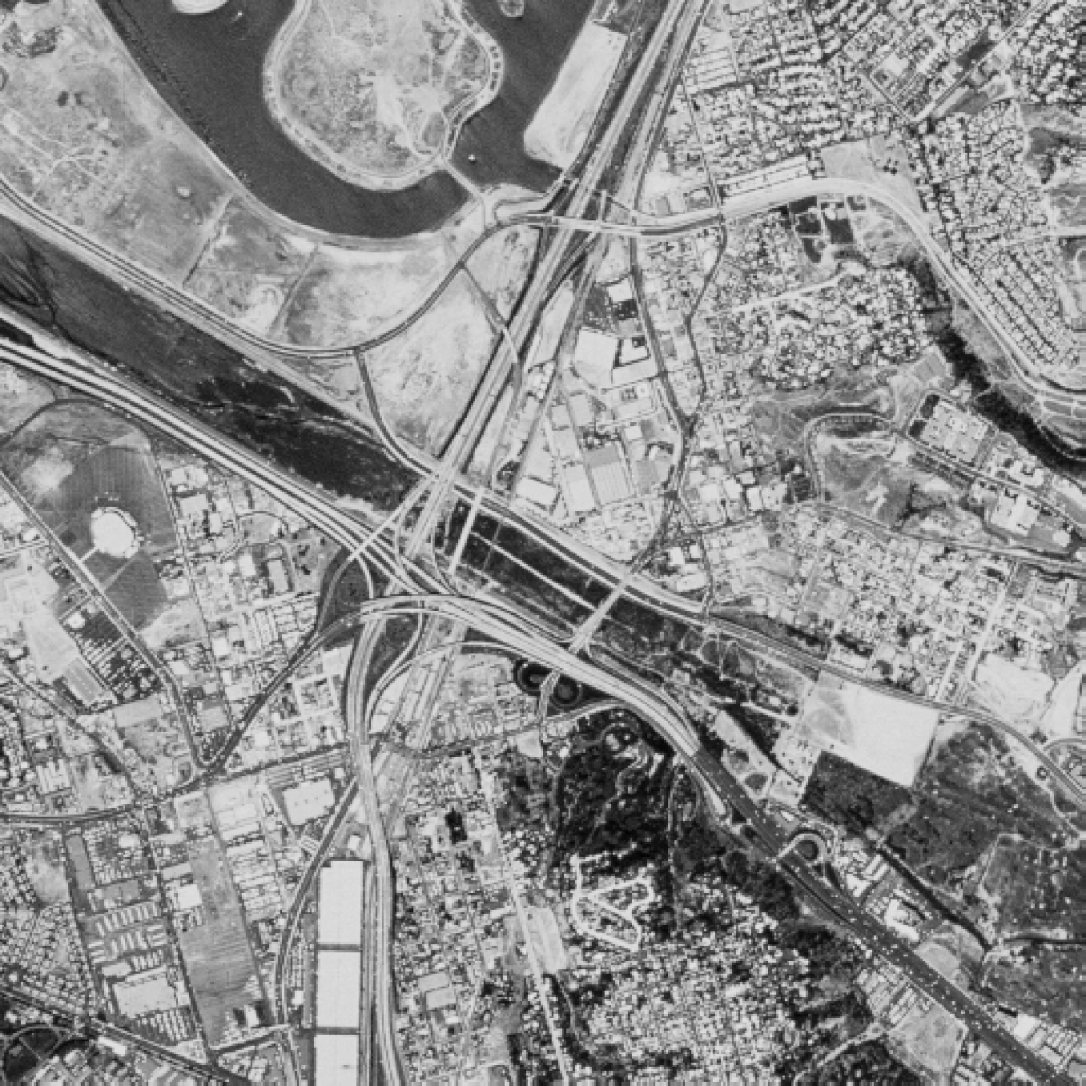
Plain grayscale San Diego aerial image.

**Figure 14:**
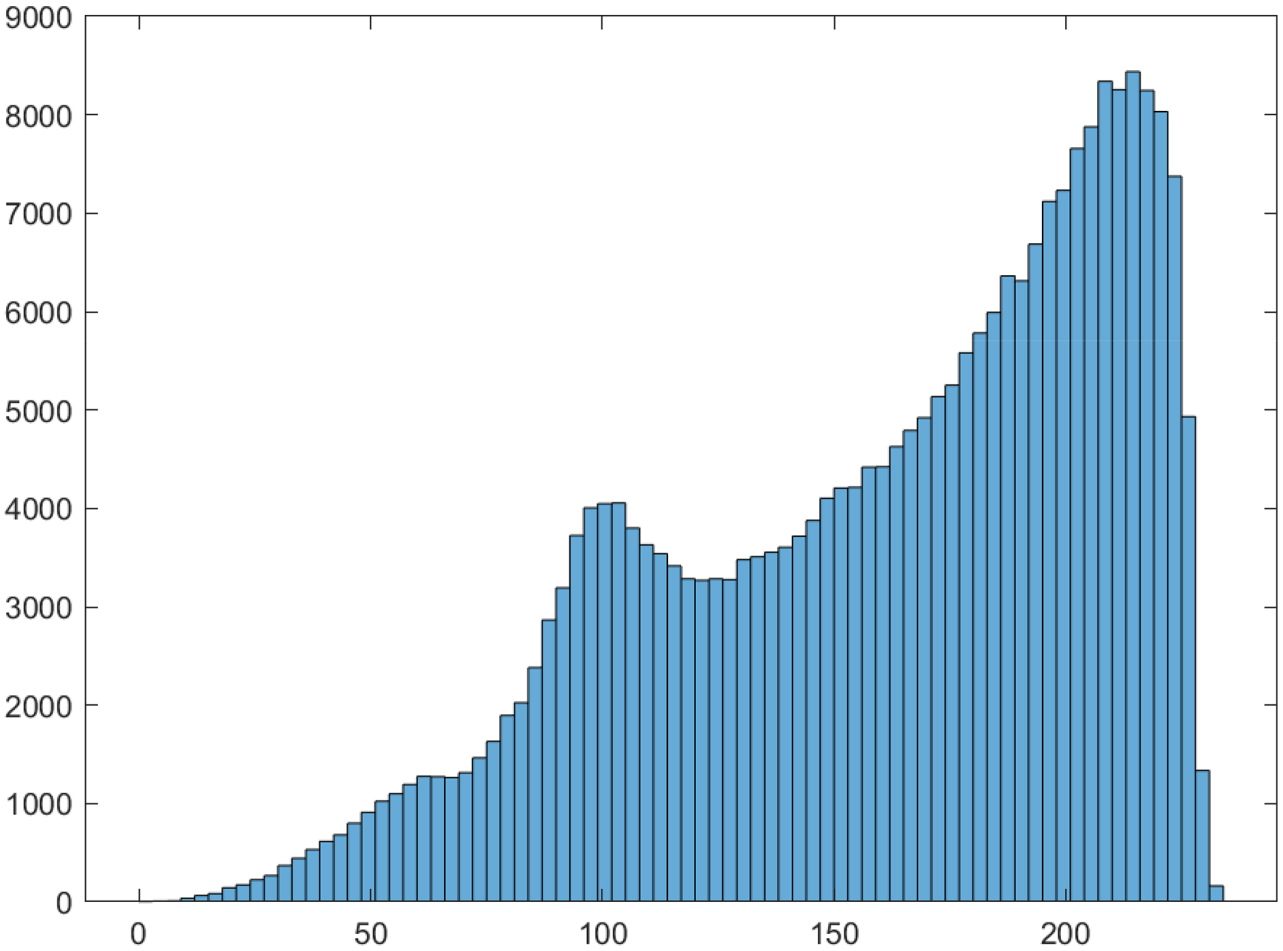
Histogram of San Diego aerial grayscale image.

**Figure 15:**
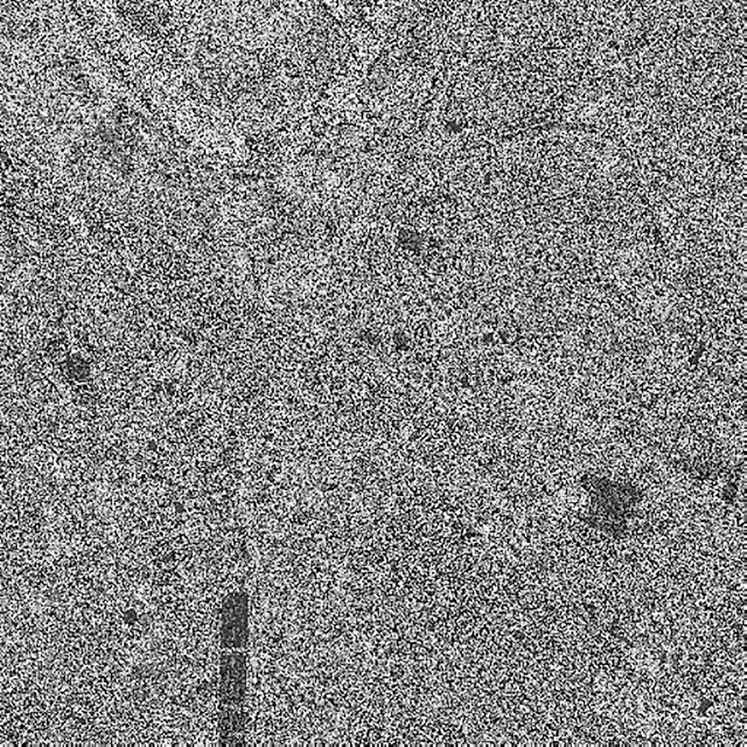
Substituted San Diego aerial image using proposed S-box.

**Figure 16:**
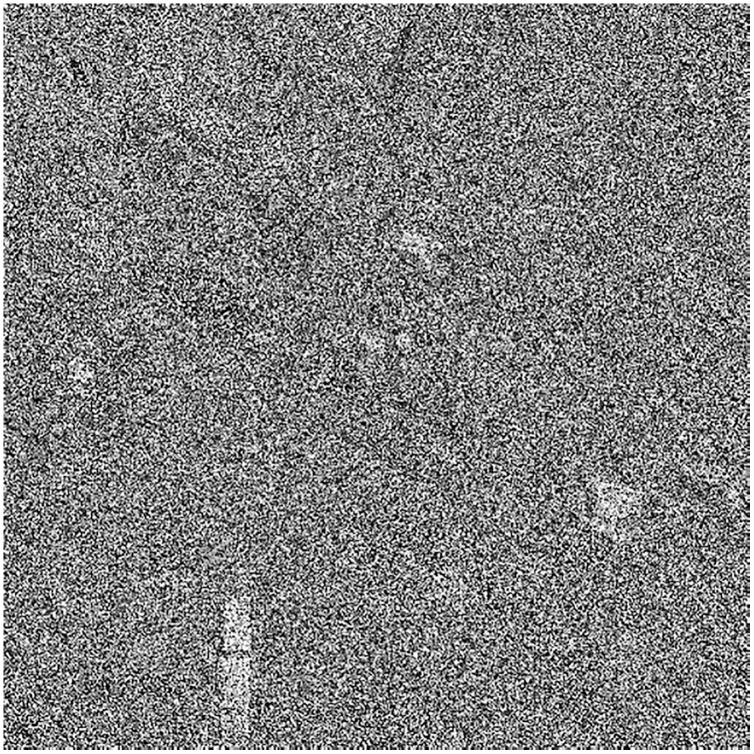
Substituted San Diego aerial image using AES S-box.

The visual demonstration of plain image (Fig. 13) substituted using proposed and AES S-box is also shown. Fig. 14 shows the histogram of plain image. The substituted image using proposed and AES S-box is shown in Figs. 15 and 16, respectively. It is evident from the figures that the proposed S-box hides all visual information contained in an image. It further justifies the effectiveness of the proposed S-box in image encryption. It can be concluded that the proposed S-box can be used as a confusion component in any application of image encryption.

## Discussion

We proposed a systematic S-box to achieve near-optimal cryptographic properties of bijective, nonlinearity, SAC, BIC, DP, and LP. The generated S-box is given in Table 1. The design assumption is to reserves engineer the attacking scenario of differential cryptanalysis, which uses the high probability of DP value to mount an attack. However, dispersion property that measures the randomness among output differentials is used within the loop to generate S-box positions systematically. It is hypothesized that high dispersion among S-box’s output differential entails improved differential uniformity. The proposed S-box achieved a maximum DP value of 0.03125, which shows the maximum value in the DDT table is 8 with 20 entries. Thus, DP value is significantly improved as compared to the existing chaos-based S-box (Nizam Chew & Ismail, 2020; Beg et al., 2020; Zahid, Arshad & Ahmad, 2019; Siddiqui, Naseer & Ehatisham-ul-Haq, 2021; Ahmad et al., 2020; Hayat, Azam & Asif, 2018; Hussain et al., 2018; Siddiqui et al., 2020). Thus, as a confusion component in the cryptosystem, the proposed systematic S-box entails strong resistance against differential cryptanalysis. The achieved cryptographic properties of our proposed S-box are summarized in Table 14.

**Table 14:** Comparison of cryptographic properties of proposed S-box.

Index	S-box	Nonlinearity		SAC		BIC (Average)	DP _max_
		Min	Max	Min	Max		
1	Proposed S-box	102	108	0.4993	0.5253	108.0	0.0312
2	Belazi & El-Latif (2017)	100	108	0.4375	0.5624	103.78	0.0390
3	Islam & Liu (2017)	102	108	0.4219	0.5938	104	0.0390
4	Özkaynak, Çelik & Özer (2017)	100	108	0.4218	0.4982	103.1	0.0390
5	Khan et al. (2018)	100	108	0.4218	0.5781	103.5	0.0312
6	Hayat, Azam & Asif (2018)	100	107	0.4063	0.5938	103.3	0.0390
7	Alzaidi et al. (2018)	110	112	0.421	0.609	104	0.0390
8	Lu, Zhu & Wang (2019)	104	108	0.3918	0.4916	104.14	0.0390
9	Zahid, Arshad & Ahmad (2019)	106	108	0.421	0.578	103.5	0.0390
10	Faheem et al. (2020)	100	106	0.4121	0.5214	103.2	0.0390
11	Wang et al. (2020)	110	112	0.4219	0.5781	104.7	0.0390
12	Ahmad et al. (2020)	112	116	0.421	0.5313	104.5	0.0468
13	Özkaynak (2020)	100	108	0.375	0.4915	104.75	0.0390
14	Nizam Chew & Ismail (2020)	112	112	0.4370	0.5468	112	0.0156
15	Zhu et al. (2020)	111	114	0.4218	0.5703	106.35	0.0390
16	Jiang & Ding (2021)	100	108	0.4746	0.5159	105	0.0390
17	Shakiba (2020)	100	104.4	0.4063	0.5017	104.25	0.0468
18	Hua et al. (2021)	102	108	0.4688	0.5938	103.21	0.0546
19	Aboytes-González et al. (2018)	112	112	0.4842	0.5013	112	0.0156
20	AES (Daemen & Vincent, 1998)	112	112	0.453	0.562	112	0.0156
21	Gray (Tran, Bui & Duong, 2008)	112	112	0.437	0.562	111.46	0.0156
22	APA (Cui & Cao, 2007)	112	112	0.437	0.562	112	0.0156

This article introduced a relatively new cryptanalysis known as a boomerang attack. The BCT table is generated to individually analyze the differential characteristics of cryptosystem system components. BCT provides more versatility than DDT and finds the nonzero pairs $({\Delta _i},{\nabla _o})$ using switching technique where DDT $({\Delta _i},{\Delta _o})=0$. The differential uniformity of our proposed S-box is 16 in BCT. Further, the FBCT of proposed S-box is also generated. FBCT is a variant of BCT for cryptosystem employing the Feistel structure. The differential uniformity, F-boomerang uniformity in FBCT, of proposed S-box is 12. The chaotic maps are inherently provided nonlinear trajectories in the real domain. Therefore, efficient mapping of chaotic domain retains the nonlinear behavior. Our proposed S-box has maximum and average nonlinearity of 108 and 106, respectively, which is well above the required bound and comparable to existing chaos-based S-boxes given in Siddiqui, Naseer & Ehatisham-ul-Haq (2021), Nizam Chew & Ismail (2020), Beg et al. (2020), Zahid, Arshad & Ahmad (2019), Farwa, Shah & Idrees (2016), Hussain et al. (2013a, 2013b), Ahmad et al. (2020), Hayat, Azam & Asif (2018), Khan, Ahmed & Saleem (2019), Aboytes-González et al. (2018), Hussain et al. (2018) and Siddiqui et al. (2020). Highly nonlinear mappings in encryption algorithms are considered vital to resist linear cryptanalysis. The proposed S-box method of dispersion-based randomization inadvertently achieves an excellent linear probability value of 0.1028, better than most existing methods (Aboytes-González et al., 2018; Siddiqui et al., 2020; Nizam Chew & Ismail, 2020; Ahmad et al., 2020; Cui & Cao, 2007; Tran, Bui & Duong, 2008). Cryptographically secure vectorial Boolean functions have avalanche and propagation properties for unpredictable substitutions. The average value of the SAC of the proposed S-box is 0.5123, which is very close to the ideal value of 0.5. Thus, we can say that our proposed S-box is highly nonlinear and behave unpredictable manner in any symmetric encryption algorithm. The proposed S-box can generate a balanced output that can be validated using the bijective property.

Further, the proposed nonlinear mapping, real to decimal, generated cryptographically secure PRN were tested using the NIST approved test suite. All of the tests in the NIST suite were passed, and *P*-values were well under the accepted range (0.01 < *P*-value < 1.00). The NIST suite’s frequency and block frequency test also validates the balance properties. We also employed our S-box in image encryption algorithm and performed various statistical tests to investigate the proposed S-box’s performance and suitability in image encryption applications. The proposed S-box shows excellent statistical entropy, energy, correlation, contrast, homogeneity.

## Conclusions

A novel method to generate a near-optimal S-box is proposed. A chaotic multilevel map is employed for initial chaotic trajectories. The given PWLCM generates cryptographically secure PRN, vetted through the NIST test. Under given design conditions, the dispersion matrix is systematically employed within the proposed design loop. The proposed design criteria efficiently substitute weak S-box positions for a robust S-box structure and near-optimal results. The proposed S-box also exhibits high dispersion in design which is critical to achieving the notion of confusion. The proposed S-boxes were evaluated based on expanded S-box design criteria. The proposed S-boxes were comparable to recently published state-of-the-art S-box designs in the field. Our results demonstrate that the proposed S-box has excellent cryptographic properties. The nonlinearity value is in the range of 100 to 108 and achieves the differential uniformity of 8. A systematic and robust methodology of chaos-based S-box is required to achieve the DP in the range of 4 to 10. The strength of our proposed S-box was also tested against new boomerang cryptanalysis. Therefore, the BCT and FBCT table of the proposed S-box was generated to find the maximum BCT/FBCT differential probability. The proposed S-box had a maximum BCT and FBCT differential probability of 0.0625 and 0.0468, respectively. The BCT/FBCT analysis provides a new insight to design and analyze the S-box for cryptosystem. Our proposed S-box shows an upper-bound value of LP of 0.1028. It is evident from the results presented in this paper that our S-box achieves an upper bound of cryptographic properties. To validate the suitability of the proposed S-box as a confusion component in image encryption algorithms, a substitution-based statistical test of entropy, energy, correlation, contrast, and homogeneity was performed to achieve the values of 7.358, 0.016, and 0.033, 10.11, 0.406, respectively. Our S-box show excellent performance against these tests and is suitable for image encryption applications.

In the future, this work can be extended to design key-based S-boxes. The S-boxes are based on chaotic parameters, where the S-boxes are dynamically generated in each round of encryption to obtain a more secure cryptosystem. Further, the differential uniformity of BCT/FBCT of chaos-based S-boxes will be analyzed to study the resistance against boomerang attack. Furthermore, the applications of the proposed S-boxes in image encryption and watermarking can be investigated.

## Supplemental Information

10.7717/peerj-cs.940/supp-1 Supplemental Information 1 SAC offset values of proposed S-box.

10.7717/peerj-cs.940/supp-2 Supplemental Information 2 Comparison of numerical results achieved of DP for the proposed S-boxes and other well-known existing S-boxes.

10.7717/peerj-cs.940/supp-3 Supplemental Information 3 Source code for majority logic image encryption.

10.7717/peerj-cs.940/supp-4 Supplemental Information 4 Source code of main technique of byte substitution.

10.7717/peerj-cs.940/supp-5 Supplemental Information 5 LAT 256* 256: generation value as: LAT=(2^n^- 2*LAT).

10.7717/peerj-cs.940/supp-6 Supplemental Information 6 FBCT source code in PYTHON.

10.7717/peerj-cs.940/supp-7 Supplemental Information 7 BCT source code in PYTHON.

## Additional Information and Declarations

### Data Availability

The following information was supplied regarding data availability: The source code files are available in the Supplemental Files. The raw data is the standard image of San Diego 2.1.02.tiff, available from: https://sipi.usc.edu/database/database.php?volume=aerials.

## References

[ref-2] Aboytes-González JA, Murguía JS, Mejía-Carlos M, González-Aguilar H, Ramírez-Torres MT (2018). Design of a strong S-box based on a matrix approach. Nonlinear Dynamics.

[ref-3] Açikkapi MŞ, Özkaynak F, Özer AB (2019). Side-channel analysis of chaos-based substitution box structures. IEEE Access.

[ref-4] Ahmad M, Al-Solami E, Alghamdi AM, Yousaf MA (2020). Bijective S-boxes method using improved chaotic map-based heuristic search and algebraic group structures. IEEE Access.

[ref-5] Ahmad M, Alauddin M, AlSharari HD (2018). Heuristic approach for nonlinear n × n (3 ≤ n ≤ 7) substitution-boxes. Advances in Intelligent Systems and Computing.

[ref-6] Ahmed HA, Zolkipli MF, Ahmad M (2019). A novel efficient substitution-box design based on firefly algorithm and discrete chaotic map. Neural Computing and Applications.

[ref-8] Alhadawi HS, Majid MA, Lambić D, Ahmad M (2021). A novel method of s-box design based on discrete chaotic maps and cuckoo search algorithm. Multimedia Tools and Applications.

[ref-9] Alzaidi AA, Ahmad M, Doja MN, Solami EA, Beg MMS (2018). A new 1D chaotic map and β-Hill climbing for generating substitution-boxes. IEEE Access.

[ref-10] Aoki K, Ichikawa T, Kanda M, Matsui M, Moriai S, Nakajima J, Tokita T (2001). Camellia: a 128-bit block cipher suitable for multiple platforms—design and analysis, selected areas in cryptography.

[ref-11] Artuğer F, Özkaynak F (2020). A novel method for performance improvement of chaos-based substitution boxes. Symmetry.

[ref-12] Avaroʇlu E, Tuncer T, Özer AB, Türk M (2014). A new method for hybrid pseudo random number generator. Informacije MIDEM.

[ref-13] Azam NA, Hayat U, Ullah I (2018). An injective s-box design scheme over an ordered isomorphic elliptic curve and its characterization. Security and Communication Networks.

[ref-14] Beg S, Ahmad N, Anjum A, Ahmad M, Khan A, Baig F, Khan A (2020). S-box design based on optimize LFT parameter selection: a practical approach in recommendation system domain. Multimedia Tools and Applications.

[ref-15] Behnia S, Akhavan A, Akhshani A, Samsudin A (2011). A novel dynamic model of pseudo random number generator. Journal of Computational and Applied Mathematics.

[ref-16] Belazi A, El-Latif AAA (2017). A simple yet efficient S-box method based on chaotic sine map. Optik.

[ref-17] Biham E, Shamir A (1991). Differential cryptanalysis of DES-like cryptosystems. Journal of Cryptology.

[ref-18] Biryukov A, van Tilborg HCA, Jajodia S (2011). Chosen ciphertext attack. Encyclopedia of Cryptography and Security.

[ref-19] Biryukov A, Perrin L (2015). On reverse-engineering S-boxes with hidden design criteria or structure, advances in cryptology – CRYPTO 2015.

[ref-20] Bogdanov A, Knudsen LR, Leander G, Paar C, Poschmann A, Robshaw MJB, Seurin Y, Vikkelsoe C (2007). PRESENT: an ultra-lightweight block cipher, cryptographic hardware and embedded systems - CHES 2007.

[ref-21] Boukerrou H, Huynh P, Lallemand V, Mandal B, Minier M (2020). On the feistel counterpart of the boomerang connectivity table: introduction and analysis of the FBCT. IACR Transactions on Symmetric Cryptology.

[ref-22] Boura C, Canteaut A (2018). On the boomerang uniformity of cryptographic Sboxes. IACR Transactions on Symmetric Cryptology.

[ref-23] Chen G, Chen Y, Liao X (2007). An extended method for obtaining S-boxes based on three-dimensional chaotic baker maps. Chaos Solitons and Fractals.

[ref-24] Chen S-L, Chang S-M, Lin W-W, Hwang T (2008). Digital secure-communication using robust hyper-chaotic systems. International Journal of Bifurcation and Chaos.

[ref-25] Cid C, Huang T, Peyrin T, Sasaki Y, Song L (2018). Boomerang connectivity table: a new cryptanalysis tool.

[ref-26] Cui L, Cao Y (2007). A new S-box structure named affine-power-affine. International Journal of Innovative Computing, Information and Control.

[ref-27] Daemen J, Rijmen V (2002). The design of Rijndael - the advanced encryption standard Information Security and Cryptography.

[ref-28] Daemen J, Vincent R (1998). AES submission document on Rijndael. https://csrc.nist.gov/csrc/media/projects/cryptographic-standards-and-guidelines/documents/aes-development/rijndael-ammended.pdf.

[ref-29] Dawson MH, Tavares SE (1991). An expanded set of S-box design criteria based on information theory and its relation to differential-like attacks.

[ref-30] Dimitrov MM (2020). On the design of chaos-based S-boxes. IEEE Access.

[ref-31] EL-Latif AAA, Abd-El-Atty B, Venegas-Andraca SE (2019). A novel image steganography technique based on quantum substitution boxes. Optics & Laser Technology.

[ref-1] ETSI (2001). 3rd Generation Partnership Project; Technical Specification Group Services and System Aspects; 3G Security; Specification of the 3GPP Confidentiality and Integrity Algorithms; Document 2: KASUMI Specification, V 31.1. https://portal.etsi.org/new3g/tb/other/algorithms/35202-311.pdf.

[ref-32] Faheem ZB, Ali A, Khan MA, Ul-Haq ME, Ahmad W (2020). Highly dispersive substitution box (S-Box) design using chaos. ETRI Journal.

[ref-33] Farah T, Rhouma R, Belghith S (2017). A novel method for designing S-box based on chaotic map and teaching-learning-based optimization. Nonlinear Dynamics.

[ref-34] Farhan AK, Ali RS, Natiq H, Al-Saidi NMG (2019). A new S-box generation algorithm based on multistability behavior of a plasma perturbation model. IEEE Access.

[ref-35] Farwa S, Muhammad N, Shah T, Ahmad S (2017). A novel image encryption based on algebraic S-box and Arnold transform. 3D Research.

[ref-36] Farwa S, Shah T, Idrees L (2016). A highly nonlinear S-box based on a fractional linear transformation. SpringerPlus.

[ref-38] Gangadari BR, Ahamed SR, Mahapatra R, Sinha RK (2015). Design of cryptographically secure AES S-box using cellular automata.

[ref-39] Handschuh H, van Tilborg HCA, Jajodia S (2011). RC6. Encyclopedia of Cryptography and Security.

[ref-40] Hayat U, Azam NA, Asif M (2018). A method of generating 8 × 8 substitution boxes based on elliptic curves. Wireless Personal Communications.

[ref-41] Heys HM (2002). A tutorial on linear and differential cryptanalysis. Cryptologia.

[ref-42] Hong S, Lee S, Lim J, Sung J, Cheon DH, Cho I (2000). Provable security against differential and linear cryptanalysis for the SPN structure. Fast Software Encryption.

[ref-43] Hua Z, Li J, Chen Y, Yi S (2021). Design and application of an S-box using complete latin square. Nonlinear Dynamics.

[ref-44] Hussain I, Anees A, Al-Maadeed TA, Mustafa MT (2019). Construction of S-box based on chaotic map and algebraic structures. Symmetry.

[ref-45] Hussain I, Anees A, Aslam M, Ahmed R, Siddiqui N (2018). A noise resistant symmetric key cryptosystem based on S8 S-boxes and chaotic maps. European Physical Journal Plus.

[ref-46] Hussain I, Shah T, Gondal MA, Khan WA, Mahmood H (2013a). A group theoretic approach to construct cryptographically strong substitution boxes. Neural Computing and Applications.

[ref-47] Hussain I, Shah T, Gondal MA, Mahmood H (2012). Generalized majority logic criterion to analyze the statistical strength of S-boxes. Zeitschrift Für Naturforschung A.

[ref-48] Hussain I, Shah T, Mahmood H, Gondal MA (2013b). A projective general linear group based algorithm for the construction of substitution box for block ciphers. Neural Computing and Applications.

[ref-49] Irfan M, Ali A, Khan MA, Ehatisham-ul-Haq M, Mehmood Shah SN, Saboor A, Ahmad W (2020). Pseudorandom Number Generator (PRNG) Design Using Hyper-Chaotic Modified Robust Logistic Map (HC-MRLM). Electronics.

[ref-50] Islam F, Liu G (2017). Designing S-box based on 4D-4wing hyperchaotic system. 3D Research.

[ref-51] Jakimoski G, Kocarev L (2001). Chaos and cryptography: block encryption ciphers based on chaotic maps. IEEE Transactions on Circuits and Systems I: Fundamental Theory and Applications.

[ref-52] Jamal SS, Khan MU, Shah T (2016). A watermarking technique with chaotic fractional S-box transformation. Wireless Personal Communications.

[ref-53] Jiang Z, Ding Q (2021). Construction of an S-box based on chaotic and bent functions. Symmetry.

[ref-74] Khan MA, Khan UA, Ali A, Hussain F, Nisar MW (2019). A robust color image watermarking scheme using chaos for copyright protection. Mehran University Research Journal of Engineering and Technology.

[ref-55] Khan M, Jamal SS (2021). Lightweight chaos-based nonlinear component of block ciphers. Wireless Personal Communications.

[ref-56] Khan MA, Ali A, Jeoti V, Manzoor S (2018). A chaos-based substitution box (S-Box) design with improved differential approximation probability (DP). Iranian Journal of Science and Technology - Transactions of Electrical Engineering.

[ref-57] Khan MA, Jeoti V, Manzoor RS (2012). Performance evaluation of seed based random (SBR) interleaver in Rayleigh fading channel.

[ref-58] Khan MF, Ahmed A, Saleem K (2019). A novel cryptographic substitution box design using gaussian distribution. IEEE Access.

[ref-59] Kocarev L (2001). Chaos-based cryptography: a brief overview. IEEE Circuits and Systems Magazine.

[ref-60] Koyuncu İ, Turan Özcerit A (2017). The design and realization of a new high speed FPGA-based chaotic true random number generator. Computers and Electrical Engineering.

[ref-61] Lambić D (2018). S-box design method based on improved one-dimensional discrete chaotic map. Journal of Information and Telecommunication.

[ref-62] Langfordl SK, Hellman ME (1994). Differential-linear cryptanalysis. LNCS.

[ref-63] Li-Jiang Y, Tian-Lun C (2002). Application of chaos in genetic algorithms. Communications in Theoretical Physics.

[ref-64] Liu B, Xiang H, Liu L (2020). Reducing the dynamical degradation of digital chaotic maps with time-delay linear feedback and parameter perturbation. Mathematical Problems in Engineering.

[ref-65] Liu Q, Li P, Zhang M, Sui Y, Yang H (2015). A novel image encryption algorithm based on chaos maps with Markov properties. Communications in Nonlinear Science and Numerical Simulation.

[ref-66] Lu Q, Zhu C, Wang G (2019). A novel S-box design algorithm based on a new compound chaotic system. Entropy.

[ref-67] Magsi H, Sodhro AH, Al-Rakhami MS, Zahid N, Pirbhulal S, Wang L (2021). A novel adaptive battery-aware algorithm for data transmission in IoT-based healthcare applications. Electronics.

[ref-68] Masood F, Driss M, Boulila W, Ahmad J, Rehman SU, Jan SU, Qayyum A, Buchanan WJ (2021). A lightweight chaos-based medical image encryption scheme using random shuffling and XOR operations. Wireless Personal Communications.

[ref-69] Matsui M, Gollmann D (1996). New structure of block ciphers with provable security against differential and linear cryptanalysis. Fast Software Encryption. FSE 1996. Lecture Notes in Computer Science.

[ref-70] Meier W, Staffelbach O, Quisquater JJ, Vandewalle J (1990). Nonlinearity criteria for cryptographic functions. Advances in Cryptology — EUROCRYPT ’89. EUROCRYPT 1989. Lecture Notes in Computer Science.

[ref-71] Miroslaw S, Seredynski F (2011). Designing cryptographically strong S-boxes with use of 1d cellular automata. Journal of Cellular Automata.

[ref-72] Mohananthini N, Mohamed Parvees MY, Abdul Samath J (2021). Lightweight image encryption: a chaotic ARX block cipher. Journal of Circuits, Systems and Computers.

[ref-73] Mondal B, Mandal T (2017). A light weight secure image encryption scheme based on chaos & DNA computing. Journal of King Saud University - Computer and Information Sciences.

[ref-75] Murillo-Escobar MA, Cruz-Hernández C, Abundiz-Pérez F, López-Gutiérrez RM, Acosta Del Campo OR (2015). A RGB image encryption algorithm based on total plain image characteristics and chaos. Signal Processing.

[ref-76] National Institute of Standards and Technology (1999). Data Encryption Standard (DES). Federal Information Processing Standards Publication (FIPS PUB 46-3).

[ref-37] National Institute of Standards and Technology (2001). Advanced Encryption Standard (AES), National Institute of Standards and Technology, U.S. Department of Commerce. https://nvlpubs.nist.gov/nistpubs/FIPS/NIST.FIPS.197.pdf.

[ref-77] Nizam Chew LC, Ismail ES (2020). S-box construction based on linear fractional transformation and permutation function. Symmetry.

[ref-78] Nyberg K, Davies DW (1991). Perfect nonlinear S-boxes. Advances in Cryptology — EUROCRYPT ’91. EUROCRYPT 1991. Lecture Notes in Computer Science.

[ref-79] Özkaynak F (2019). An analysis and generation toolbox for chaotic substitution boxes: a case study based on Chaotic Labyrinth Rene Thomas system. Iranian Journal of Science and Technology - Transactions of Electrical Engineering.

[ref-80] Özkaynak F (2017). Construction of robust substitution boxes based on chaotic systems. Neural Computing and Applications.

[ref-81] Özkaynak F (2020). On the effect of chaotic system in performance characteristics of chaos based S-box designs. Physica A: Statistical Mechanics and Its Applications.

[ref-82] Özkaynak F, Çelik V, Özer AB (2017). A new S-Box construction method based on the fractional-order chaotic Chen system. Signal Image and Video Processing.

[ref-83] Paar C, Pelzl J (2009). Understanding cryptography: a textbook for students and practitioners.

[ref-84] Pak C, Kim J, An K, Kim C, Kim K, Pak C (2019). A novel color image LSB steganography using improved 1D chaotic map. Multimedia Tools and Applications.

[ref-85] Pareek NK, Patidar V, Sud KK (2006). Image encryption using chaotic logistic map. Image and Vision Computing.

[ref-86] Picek S, Mariot L, Yang B, Jakobovic D, Mentens N (2017). Design of S-boxes defined with cellular automata rules.

[ref-87] Picek Stjepan, Batina L, Jakobović D, Ege B, Golub M, Naccache D, Sauveron D (2014). S-box, set, match: a toolbox for S-box analysis. Information Security Theory and Practice. Securing the Internet of Things. WISTP 2014. Lecture Notes in Computer Science.

[ref-88] Prathiba A, Bhaaskaran VSK (2018). Lightweight S-box architecture for secure internet of things. Information-an International Interdisciplinary Journal.

[ref-89] Rajendran S, Doraipandian M (2021). Chaos based secure medical image transmission model for IoT - powered healthcare systems. IOP Conference Series: Materials Science and Engineering.

[ref-90] Rezk AA, Madian AH, Radwan AG, Soliman AM (2019). Reconfigurable chaotic pseudo random number generator based on FPGA. AEU-International Journal of Electronics and Communications.

[ref-91] Rivest RL (1995). The RC5 encryption algorithm.

[ref-92] Schneier B (1993). Description of a new variable-length key, 64-bit block cipher (Blowfish).

[ref-93] Seredynski F, Bouvry P, Zomaya AY (2004). Cellular automata computations and secret key cryptography. Parallel Computing.

[ref-94] Shah T, Hussain I, Gondal MA, Mahmood H (2011). Statistical analysis of S-box in image encryption applications based on majority logic criterion.

[ref-95] Shakiba A (2020). Generating dynamical S-boxes using 1D Chebyshev chaotic maps. Journal of Computing and Security.

[ref-96] Shannon CE (1949). Communication theory of secrecy systems. Bell System Technical Journal.

[ref-97] Siddiqui N, Naseer A, Ehatisham-ul-Haq M (2021). A novel scheme of substitution-box design based on modified Pascal’s triangle and elliptic curve. Wireless Personal Communications.

[ref-98] Siddiqui N, Yousaf F, Murtaza F, Ehatisham-ul-Haq M, Ashraf MU, Alghamdi AM, Alfakeeh AS (2020). A highly nonlinear substitution-box (S-Box) design using action of modular group on a projective line over a finite field. PLOS ONE.

[ref-99] Siddiqui TJ, Khare A (2021). Chaos-based video steganography method in discrete cosine transform domain. International Journal of Image and Graphics.

[ref-100] Singh P, Raman B (2017). A secured robust watermarking scheme based on majority voting concept for rightful ownership assertion. Multimedia Tools and Applications.

[ref-101] Solami Eal, Ahmad M, Volos C, Doja MN, Beg MMS (2018). A new hyperchaotic system-based design for efficient bijective substitution-boxes. Entropy.

[ref-102] Song L, Qin X, Hu L (2019). Boomerang connectivity table revisited: application to SKINNY and AES. IACR Transactions on Symmetric Cryptology.

[ref-103] Standaert FX, Piret G, Quisquater JJ (2003). Cryptanalysis of block ciphers: a survey.

[ref-104] Szaban M, Seredynski F (2012). , Dynamic cellular automata-based S-boxes.

[ref-105] Tang G, Liao X, Chen Y (2005). A novel method for designing S-boxes based on chaotic maps. Chaos Solitons and Fractals.

[ref-106] Tanyildizi E, Özkaynak F (2019). A new chaotic S-box generation method using parameter optimization of one dimensional chaotic maps. IEEE Access.

[ref-107] Tian Y, Lu Z (2017). Chaotic S-box: intertwining logistic map and bacterial foraging optimization. Mathematical Problems in Engineering.

[ref-108] Tian Y, Lu Z (2016). S-box: six-dimensional compound hyperchaotic map and artificial bee colony algorithm. Journal of Systems Engineering and Electronics.

[ref-109] Tran MT, Bui DK, Duong AD (2008). Gray S-box for advanced encryption standard.

[ref-110] Wagner D (1999). The boomerang attack.

[ref-111] Wang X, Çavuşoğlu Ü, Kacar S, Akgul A, Pham V-T, Jafari S, Alsaadi FE, Nguyen XQ (2019). S-Box based image encryption application using a chaotic system without equilibrium. Applied Sciences.

[ref-112] Wang Y, Xie Q, Wu Y, Du B (2009). A software for S-box performance analysis and test.

[ref-113] Wang Y, Zhang Z, Zhang LY, Feng J, Gao J, Lei P (2020). A genetic algorithm for constructing bijective substitution boxes with high nonlinearity. Information Sciences.

[ref-7] Weber AE (1981). San Diego Aerial Image 2.1.02.Tiff. https://sipi.usc.edu/database/database.php?volume=aerials&image=2#top.

[ref-115] Webster AF, Tavares SE, Williams HC (1986). On the design of S-boxes. Advances in Cryptology – CRYPTO ’85 Proceedings. CRYPTO 1985. Lecture Notes in Computer Science.

[ref-117] Yavuz E, Yazıcı R, Kasapbaşı MC, Yamaç E (2016). A chaos-based image encryption algorithm with simple logical functions. Computers & Electrical Engineering.

[ref-118] Yi L, Tong X, Wang Z, Zhang M, Zhu H, Liu J (2019). A novel block encryption algorithm based on chaotic S-box for wireless sensor network. IEEE Access.

[ref-119] Yi X, Cheng S, You X (1997). A method for obtaining cryptographically strong 8×8 S-boxes.

[ref-120] Zahid AH, Arshad MJ, Ahmad M (2019). A novel construction of efficient substitution-boxes using cubic fractional transformation. Entropy.

[ref-121] Zahid AH, Iliyasu AM, Ahmad M, Shaban MMU, Arshad MJ, Alhadawi HS, El-Latif AAA (2021). A novel construction of dynamic S-box with high nonlinearity using heuristic evolution. IEEE Access.

[ref-122] Zamli KZ (2021). Optimizing S-box generation based on the adaptive agent heroes and cowards algorithm. Expert Systems with Applications.

[ref-123] Zhu H, Tong X, Wang Z, Ma J (2020). A novel method of dynamic S-box design based on combined chaotic map and fitness function. Multimedia Tools and Applications.

