# Supplementary material for: A novel systematic byte substitution method to design strong bijective substitution box (S-box) using piece-wise-linear chaotic map
Source: PeerJ Comput Sci. 2022 May 11;8:e940. doi: 10.7717/peerj-cs.940 (PMC9138039; doi:10.7717/peerj-cs.940)
Supplement: Supplemental Information 1 [file peerj-cs-08-940-s001.docx]

| Index | 1 | 2 | 3 | 4 | 5 | 6 | 7 | 8 |
| --- | --- | --- | --- | --- | --- | --- | --- | --- |
| 1 | 0.046 | 0.015 | 0 | 0.015 | 0.015 | 0.015 | 0.062 | 0.062 |
| 2 | 0.046 | 0.046 | 0.03 | 0.093 | 0.015 | 0.015 | 0 | 0.078 |
| 3 | 0.015 | 0.093 | 0.031 | 0.093 | 0.031 | 0 | 0.031 | 0.031 |
| 4 | 0 | 0.015 | 0.031 | 0.031 | 0.031 | 0.062 | 0.062 | 0.062 |
| 5 | 0.109 | 0.046 | 0 | 0.046 | 0.046 | 0.015 | 0.015 | 0.015 |
| 6 | 0.031 | 0.015 | 0.062 | 0.062 | 0 | 0.062 | 0.015 | 0.046 |
| 7 | 0.093 | 0.093 | 0.015 | 0.062 | 0.031 | 0.015 | 0.046 | 0.078 |
| 8 | 0.031 | 0.031 | 0 | 0.046 | 0.015 | 0.015 | 0.015 | 0.031 |
