# Supplementary material for: A novel systematic byte substitution method to design strong bijective substitution box (S-box) using piece-wise-linear chaotic map
Source: PeerJ Comput Sci. 2022 May 11;8:e940. doi: 10.7717/peerj-cs.940 (PMC9138039; doi:10.7717/peerj-cs.940)
Supplement: Supplemental Information 2 [file peerj-cs-08-940-s002.docx]

| **Index** | **S-box** | **DP _max_** |
| --- | --- | --- |
| 1 | Proposed initial S-box | 0.390 |
| 2 | Proposed improved S-box | 0.0312 |
| 3 | Belazi et. al. (2017)(Belazi et al., 2017) | 0.0390 |
| 4 | Islam et. al. (2017)(Islam et al., 2017) | 0.0390 |
| 5 | Özkaynak et. al. (2017)(Özkaynak, 2017) | 0.0390 |
| 6 | Khan et. al. (2018)(Khan et al., 2018) | 0.0312 |
| 7 | Azam et. al. (2018) (Hayat et al., 2018) | 0.390 |
| 8 | Alzaidi et. al. (2018) (Alzaidi et al., 2018) | 0.0390 |
| 9 | Wang et. al. (2019)(Lu et al., 2019) | 0.0390 |
| 10 | Zahid et. al. (2019)(Zahid et al., 2019) | 0.0390 |
| 11 | Faheem et. al. (2020)(bin Faheem et al., 2020) | 0.0390 |
| 12 | Wang et. al.(2020)(Wang et al., 2020) | 0.0390 |
| 13 | Ahmad et. al.(2020)(Ahmad et al., 2020) | 0.0468 |
| 14 | Özkaynak et. al.(2020) (Özkaynak, 2020) | 0.0390 |
| 15 | Chew et. al. 2020 (Nizam Chew et al., 2020) | 0.0156 |
| 16 | Zhu et. al. (2020) (Zhu et al., 2020) | 0.0390 |
| 17 | Jiang et. al.(2021) (Jiang et al., 2021) | 0.0390 |
| 18 | Shakiba et. al.(2021)(Shakiba, 2020) | 0.0468 |
| 19 | Hua et. al.(2021)(Hua et al., 2021) | 0.546 |
| 20 | Gonzalez et. al(2018) | 0.0156 |
| 21 | AES (Daemen et al., 1998) | 0.0156 |
| 22 | Gray(Tran et al., 2008) | 0.0156 |
| 23 | APA (Cui et al., 2007) | 0.0156 |
