# Supplementary figures and images for: A novel systematic byte substitution method to design strong bijective substitution box (S-box) using piece-wise-linear chaotic map
Source: PeerJ Comput Sci. 2022 May 11;8:e940. doi: 10.7717/peerj-cs.940 (PMC9138039; doi:10.7717/peerj-cs.940)

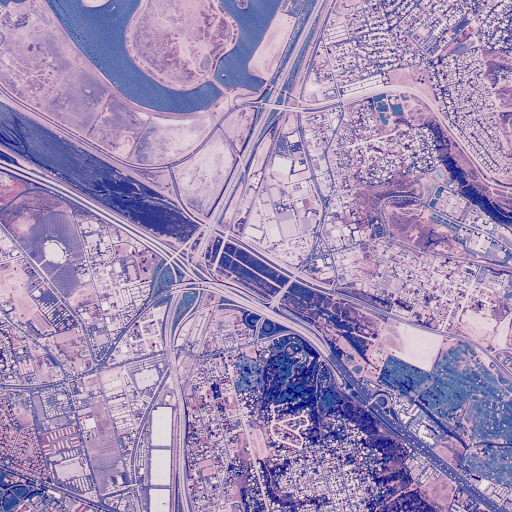

Supplement: Supplemental Information 3 [file peerj-cs-08-940-s003.zip › cs-66883-Code/2.1.02.tiff]
